# Poxviruses Bearing DNA Polymerase Mutations Show Complex Patterns of Cross-Resistance

**DOI:** 10.3390/biomedicines10030580

**Published:** 2022-03-01

**Authors:** Graciela Andrei, Pierre Fiten, Marcela Krečmerová, Ghislain Opdenakker, Dimitrios Topalis, Robert Snoeck

**Affiliations:** 1 Laboratory of Virology and Chemotherapy, Department of Microbiology, Immunology, and Transplantation, Rega Institute for Medical Research, KU Leuven, Herestraat 49, Box 1030, 3000 Leuven, Belgium; dimitri.topalis@gmail.com (D.T.); robert.snoeck@kuleuven.be (R.S.); 2 Laboratory of Immunobiology, Department of Microbiology, Immunology, and Transplantation, Rega Institute for Medical Research, KU Leuven, Herestraat 49, Box 1044, 3000 Leuven, Belgium; pierre.fiten@kuleuven.be (P.F.); ghislain.opdenakker@kuleuven.be (G.O.); 3 Institute of Organic Chemistry and Biochemistry, Czech Academy of Sciences, Flemingovo Nám. 2, 166 10 Prague, Czech Republic; marcela.krecmerova@uochb.cas.cz

**Keywords:** vaccinia virus, DNA polymerase, drug resistance, nucleotide analogues, cidofovir, phosphonoacetic acid

## Abstract

Despite the eradication of smallpox four decades ago, poxviruses continue to be a threat to humans and animals. The arsenal of anti-poxvirus agents is very limited and understanding mechanisms of resistance to agents targeting viral DNA polymerases is fundamental for the development of antiviral therapies. We describe here the phenotypic and genotypic characterization of poxvirus DNA polymerase mutants isolated under selective pressure with different acyclic nucleoside phosphonates, including HPMPC (cidofovir), cHPMPC, HPMPA, cHPMPA, HPMPDAP, HPMPO-DAPy, and PMEO-DAPy, and the pyrophosphate analogue phosphonoacetic acid. Vaccinia virus (VACV) and cowpox virus drug-resistant viral clones emerging under drug pressure were characterized phenotypically (drug-susceptibility profile) and genotypically (DNA polymerase sequencing). Different amino acid changes in the polymerase domain and in the 3′-5′ exonuclease domain were linked to drug resistance. Changes in the 3′-5′ domain emerged earlier than in the polymerase domain when viruses acquired a combination of mutations. Our study highlights the importance of poxvirus DNA polymerase residues 314, 613, 684, 688, and 851, previously linked to drug resistance, and identified several novel mutations in the 3′-5′ exonuclease domain (M313I, F354L, D480Y) and in the DNA polymerase domain (A632T, T831I, E856K, L924F) associated with different drug-susceptibility profiles. Furthermore, a combination of mutations resulted in complex patterns of cross-resistance. Modeling of the VACV DNA polymerase bearing the newly described mutations was performed to understand the effects of these mutations on the structure of the viral enzyme. We demonstrated the emergence of drug-resistant DNA polymerase mutations in complex patterns to be considered in case such mutations should eventually arise in the clinic.

## 1. Introduction

Smallpox, caused by variola virus (VARV), has been one of the deadliest diseases in human history, causing millions of deaths. Global smallpox eradication was possible thanks to a program started by the World Health Organization in 1959 and an Intensified Eradication Program began in 1967 based on mass vaccination campaigns and the establishment of a case surveillance system. The last naturally acquired case of smallpox was diagnosed on 30 October 1977, and the last person who died of smallpox was recorded on 11 September 1978. On 26 October 1979, the World Health Organization WHO announced the eradication of smallpox, which was officially declared on 8 May 1980, during the 33rd World Health Assembly.

Despite smallpox eradication, there exists growing concern of the possible use of VARV as a bioweapon [1,2,3]. There are only two laboratories in the world (the Centers for Disease Control and Prevention (CDC) in the United States and the Russian State Centre for Research on Virology and Biotechnology in the Russian Federation) that are approved to use VARV for research. However, there exists credible concern that some VARV stocks may have been stored by some countries in the past and may have arrived in the hands of people with criminal intentions.

A bioterrorist smallpox attack could be especially destructive and harmful since most of today’s population lacks immunity against the virus considering that routine vaccination ended in the 1970s [4]. Vaccination alone would likely not be sufficient to respond to a smallpox bioterrorist attack as the vaccine needs to be administered within 3–5 days following infection to be effective therapeutically and disease symptoms appear about 7 to 14 days after exposure to the variola virus. Therefore, antivirals are critical to manage a potential bioterrorist attack with VARV [5]. Tecovirimat (ST-246, Tpoxx^®^) is the first antiviral agent specifically indicated for the treatment of smallpox disease in adults and pediatric patients and was approved by the US Food and Drug Administration (FDA) in 2018 [6]. Tpoxx inhibits the viral envelope formation and the egress of the virus by targeting the p37 protein of orthopoxviruses [7,8]. In 2018, brincidofovir (BCV, CMX-001, hexadecyloxypropyl-cidofovir [HDP-CDV]), a lipid conjugate of the acyclic nucleoside phosphonate (ANP) cidofovir (CDV, HPMPC), received the Orphan Drug Designation from the FDA for the treatment of smallpox [1,9]. Brincidofovir (brand name Tembexa) is a prodrug of cidofovir that was designed to release cidofovir intracellularly, resulting in higher intracellular and lower plasma concentrations of cidofovir, and effectively increasing its anti-DNA virus activity and oral bioavailability [10].

The WHO Advisory Committee on Variola Virus Research recommended pursuing research on anti-poxvirus drugs [11]. As well as the threat of an intentional release of smallpox, other poxviruses are a danger for human health. For instance, monkeypox virus (MPXV) is a zoonotic poxvirus endemic in Central and Western Africa, genetically related to VACV and VARV [12,13,14]. Furthermore, MPXV was introduced into the United States by the exotic pet trade in 2003, as well as in the United Kingdom, Singapore, and Israel in the past few years [15]. Molluscum contagiosum virus (MCV), similar to VARV, is a human-specific poxvirus; however, differently to VARV, MCV causes common, usually benign, and mild skin infections worldwide, particularly frequent in children, sexually active adults, and individuals with an impaired immune system [16,17]. Several poxviruses, such as buffalopox, vaccinia virus (VACV), and cowpox virus (CPXV), are still causing animal diseases and economic loss, and outbreaks of VACV and CPXV, responsible for cutaneous lesions in humans, have been reported [18,19,20,21,22].

In the search for promising anti-poxvirus agents, numerous compounds have been evaluated in vitro and in vivo against poxviruses. In addition to cidofovir, several ANPs have shown potent and selective activity against poxviruses [23,24,25]. ANPs, one of the most successful classes of antiviral molecules, remain a good option for developing novel broad-spectrum anti-poxvirus agents [23,26,27,28,29,30,31]. Cidofovir, similarly to other ANPs, does not require activation by viral kinases and is phosphorylated to the diphosphate active form (i.e., HPMPCpp) by cellular kinases before inhibiting polymerases. Cidofovir’s antiviral effect is the result of a selective interaction of its diphosphoryl metabolite HPMPCpp with the viral DNA polymerases, and the selectivity of this drug is explained by a higher affinity of HPMPCpp for viral DNA polymerases than for host cell polymerases, with a much higher binding affinity of HPMPCpp, as represented by the inhibition constant (Ki), for viral DNA polymerases than for human DNA polymerases. HPMPCpp can function as a competitive inhibitor of dCTP binding, and it can also act as an alternative substrate and can be incorporated into the growing DNA chain [32]. Since CDV contains a hydroxyl function in the acyclic side chain, its incorporation does not inevitably result in chain termination. Not only cidofovir but other classes of ANPs are able to inhibit poxvirus replication by targeting the virus DNA polymerase, though their mode of interaction with this viral enzyme appears to be different [33,34].

The mode of action of ANPs differs from that of the pyrophosphate analogues, such as phosphonoacetic acid (PAA), that can directly inhibit viral polymerases independently of activation by viral or cellular kinases. Pyrophosphate analogues interfere with the release of pyrophosphate from deoxynucleoside triphosphates (dNTPs) by directly targeting the dNTP-binding domain and, thus, prevent efficient binding of the incoming nucleotide [35].

The poxvirus virus DNA polymerase (E9 protein), encoded by the *E9L* gene, is a member of the B family of replicative polymerases with both polymerase and 3′-5′ exonuclease activities that are essential to support virus replication [34,36]. In addition to the E9 protein (containing the catalytic subunit of the DNA polymerase), other viral proteins are vital for the synthesis of the poxvirus genome of ∼200 kbp. Among these essential proteins are included D5 (a nucleoside triphosphate with helicase–primase activity, encoded by the *D5R* gene), D4 (a uracil DNA glycosylase, encoded by the *D4R* gene), and A20 (a non-enzymatic bridging protein binding to both E9 and D4 proteins, encoded by the *A20R* gene). The heterodimer formed by A20 and D4 acts as a processivity factor for the viral DNA polymerase. Genetic and biochemical studies identified other viral proteins also involved in the replication machinery, i.e., A50 (DNA ligase), B1 (protein kinase), G5 (endonuclease), H5 (abundant hub protein), and I3 (single-stranded DNA binding protein).

Genetic studies of poxvirus DNA polymerases have identified specific mutations in the DNA polymerase that can confer temperature sensitivity, drug resistance, and (anti)mutator phenotypes. To date, the emergence of in vitro drug resistance under the pressure of ANPs has mostly focused on cidofovir [37,38,39,40] and HPMPDAP [41,42]. Mutations both in the 3′-5′exonuclease domain and in the 5′-3′ polymerase domain have been undoubtably linked to cidofovir resistance and to resistance to other ANPs, as evidenced by the construction of recombinant viruses [37,42]. The recombinant viruses bearing the DNA polymerase mutation(s) identified following selection with HPMPC and HPMPDAP, showed complex patterns of drug resistance and drug (hyper)susceptibility to different classes of DNA polymerase inhibitors (including ANPs, arabinoside cytosine (AraC), PAA, and aphidicolin) with some mutations being linked to a mutator phenotype.

The target of ANPs is the poxvirus DNA polymerase (DNA pol), and complex patterns of poxvirus drug resistance have been found [37,41,42], indicating that different ANPs may function in unique ways. Therefore, investigating the emergence and characterization of drug resistance under selective pressure of different ANPs is fundamental to understand the interactions of specific ANPs with viral polymerases and to determine patterns of cross-resistance. Here, we provide insights into the mechanism of drug resistance and the kinetic appearance of resistance to different classes of ANPs, including various HPMP [41] derivatives, two phosphonomethoxy)alkoxy]-2,4-diaminopyrimidines, i.e., (*R*)-HPMPO-DAPy, 6-[3-hydroxy-2-(phosphonomethoxy)propoxy]-2,4-diaminopyrimidine, and PMEO-DAPy, as well as the pyrophosphate PAA.

## 2. Materials and Methods

### 2.1. Cell and Virus Culture

Human embryonic lung (HEL) fibroblasts (HEL 299, CCL-137™) were cultured in minimal essential medium (MEM) supplemented with 10% heat-inactivated fetal calf serum, 2 mM l-glutamine, and 0.3% sodium bicarbonate at 37 °C in a 5% CO2 atmosphere. VACV (strain Lederle) and CPXV (strain Brighton) were grown in HEL fibroblasts using the same medium but supplemented with 2% heat-inactivated fetal calf serum.

### 2.2. Materials

The sources of the compounds were as follows: AraC (cytosine β-D-arabinofuranoside), PAA (phosphonoacetic acid), and Aph (aphidicolin) from Sigma (St. Louis, MO, USA); HPMPC, CDV (cidofovir), (*S*)-1-[3-hydroxy-2-(phosphonomethoxypropyl)cytosine], cHPMPC (cyclic (*S*)-HPMPC), and PMEA, adefovir, 9-[2-(phosphonomethoxy)ethyl]adenine, from Gilead Sciences (Foster City, CA, USA); (*S*)-HPMPA, (S)-9-[3-hydroxy-2-(phosphonomethoxy)propyl]adenine, cHPMPA (cyclic (*S*)-HPMPA), 3-deaza-(*S*)-HPMPA, (*S*)-9-[3-hydroxy-2-(phosphonomethoxy)propyl]-3-deazaadenine}, cyclic-3-deaza-(*S*)-HPMPA (cyclic-3-deaza-HPMPA), 7-deaza-HPMPA, (*S*)-9-[3-hydroxy-2-(phosphonomethoxy)propyl]-3-deazaadenine}, c-7-deaza-HPMPA, cyclic-7-deaza-(*S*)-HPMPA, (*S*)-HPMPDAP, (*S*)-1-[3-hydroxy-2-(phosphonomethoxypropyl)-2,6-diaminopurine], (*R*)-HPMPO-DAPy, 6-[3-hydroxy-2-(phosphonomethoxy)propoxy]-2,4-diaminopyrimidine, PMEO-DAPy, 6-[2-(phosphonomethoxy)ethoxy]-2,4-diaminopyrimidine], (*S*)-HPMP-5-azaC, 1-(S)-[3-hydroxy-2-(phosphonomethoxy)propyl]-5-azacytosine, cyclic (*S*)-HPMP-5-azaC, cHPMP-5-azaC, 8-aza-(*S*)-HPMPA, and cyclic-8-aza-(*S*)-HPMPA, Dr. M. Krečmerova (Institute of Organic Chemistry and Biochemistry, Academy of Sciences of the Czech Republic, Prague).

### 2.3. Selection and Purification of Drug-Resistance Viruses

VACV virus (strain Lederle) or four plaque-purified clones (denoted VV1, VV2, VV8, and VV11), derived from the Lederle strain, were passaged repeatedly in HEL fibroblasts in the presence of increasing amounts of each compound. The starting concentration was the concentration that reduced the cytopathic effect (CPE) by 50% (50% effective concentration, EC_50_) of wild-type virus. The infected cells were cultured until development of 80–100% CPE. The viruses were then harvested and used to infect fresh cell cultures in the presence of increasing concentrations (increasing the drug concentration by 2-fold with each subsequent passage). Periodically, virus growth in drug-free medium had to be performed to restore the virus titer. The starting and ending drug concentrations used for the drug escalations are provided in Table S1. The viruses that were replicated in the presence of the end drug dose were cultured once more on HEL fibroblasts with drug-free media and then plaque-purified on HEL fibroblasts. Several viral clones for each selection procedure were then selected by plaque purification and were analyzed phenotypically and genotypically.

### 2.4. Cytopathic Effect (CPE) Reduction Assay

HEL fibroblasts were grown to confluence in 96-well microtiter plates (usually 7 days after cell seeding) and infected with virus at an input of 50 PFU per well. After virus adsorption for 2 h at 37 °C, the viral inoculum was removed, and 5-fold serial dilutions of the compounds diluted in fresh medium were added to the virus-infected cells (in duplicate). The starting drug concentrations were: 200 µg/mL (PAA), 50 µg/mL (HPMPC, cHPMPC, HPMP-5-azaC, cHPMP-5-azaC, HPMPO-DAPy, PMEO-DAPy), 20 µg/mL (HPMPA, cHPMPA, HPMPDAP, cHPMPDAP, 3-deaza-HPMPA, cyclic-3-deaza-cHPMPA, 7-deaza-HPMPA, cyclic-7-deaza-HPMPA, 8-aza-HPMPA, cyclic-8-aza-HPMPA, Aphidicolin), and 5 µg/mL (AraC). Following 2–3 days of incubation at 37 °C, the CPE in each entire well was recorded microscopically based on detectable alterations of the cell morphology as soon as it reached completion in the untreated, virus-infected cells, using a 0 to 5 scale (with 0, being no CPE; 1, ∼20% CPE; 2, 20 to 40% CPE; 3, ∼40 to 60% CPE; 4, 60 to 80%; 5, 80 to 100% CPE). The EC_50_ was defined as the drug concentration that reduced the CPE by 50% compared to the untreated controls and was calculated from a nonlinear curve fit using Prism 4.0b software.

### 2.5. Genotyping of the VACV DNA Polymerase

DNA was extracted from virus-infected HEL fibroblasts using a QIAamp blood kit according to the manufacturer’s instructions (QIAGEN). The *E9L* gene (DNA polymerase) was PCR amplified as two amplicons using primer set 1 (5′-ATAATGGTCCATACGGCTCTTCCC-3′ and 5′-TGGAGCAAATACCTTACCGCCTTC-3′) and primer set 2 (5′-AGTCATCAAGGGTCCACTGTTAAAGC-3′ and 5′-GATAAACTGAATCTAACAAAGAGCGACG-3′) (Figure S1). The PCR products were purified and sequenced using E9L-specific primers (Figure S2).

## 3. Results

### 3.1. Characterization of Drug-Resistant VACV and CPXV Emerging under Different HPMP and HPMPO-DAPy Derivatives

#### 3.1.1. VACV Resistance to Cyclic-HPMPC, HPMPA, and Cyclic-HPMPA

We have previously reported the characterization of VACV HPMPC and HPMPDAP resistant viruses using the Lederle strain [37,41]. Here, we examined the changes in the VACV DNA pol arising following selection with three related ANPs, i.e., the cyclic form of HPMPC (cHPMPC), HPMPA, and cyclic-HPMPA (cHPMPA). Following the passage of the Lederle strain under increasing drug concentrations, several viral clones were isolated and characterized genotypically (sequencing of the *E9L* gene coding for the DNA pol) and phenotypically (drug-susceptibility profile). Under pressure with HPMPA and cHPMPC, the A314T change in the 3′-5′exonuclease domain, formerly associated with HPMPC-R [37,38,39,40,41,42], was identified (Table 1). In the case of cHPMPA, the change S851Y, previously identified following selection with HPMPDAP [41], was found in combination with a mutation of unknown significance (A353V) mapping to the 3′-5′exonuclease domain.

**Figure 1:**
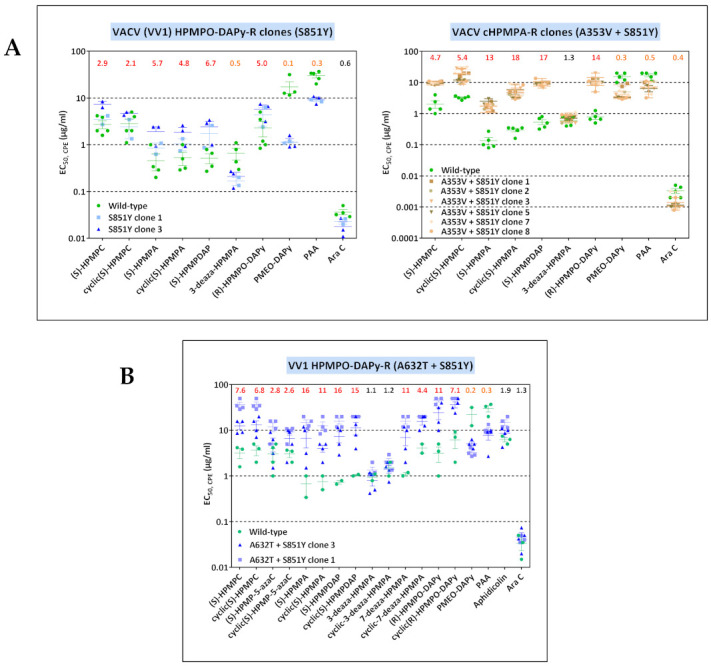
(**A**) Drug-susceptibility profile of cHPMPA-R VACV clones (A353V + S851Y) compared to VACV clones bearing the single S851Y DNA pol change recovered from VV1 HPMPO-DAPy-R #15. Drug-resistance properties of different plaque-purified viral clones bearing the single amino acid (S851Y) or double amino acid (A353V + S851Y) substitutions as determined using a CPE reduction assay with HEL fibroblasts. (**B**) Drug-susceptibility profile of selected VV1 HPMPO-DAP-R viral clones to evaluate the impact of the A632T + S851Y DNA polymerase substitutions. The A632T + A684V clones were recovered from VV1 HPMPO-DAPy-R #32. Drug-resistance properties of the different types of viral clones were established using a CPE reduction assay with HEL fibroblasts. The effects of different drugs on viruses encoding the indicated mutations were determined by calculating the EC_50_ values for the parental wild-type strain and clones bearing the specific mutations. At least two independent experiments were performed for each test compound. Horizontal lines for each drug and mutant viral clones indicate the mean values ± standard deviation. The fold resistance (ratio of the EC_50_ for the mutant viruses to the EC_50_ for the corresponding wild-type virus is marked at the top of the graph. VACV viral clones showing a ≥2-fold increase (red, bold) were considered drug resistant (R) and those with a ≤0.5-fold decrease (orange, bold) drug hypersensitive (hs).

Next, selected VACV clones were phenotyped by cytopathic effect (CPE) reduction assay in HEL fibroblasts. Different classes of ANPs were used, including HPMP derivatives ((*S*)-HPMPC, cyclic(*S*)-HPMPC, (*S*)-HPMP-5azaC, cyclic(*S*)-HPMP-5-azaC, (*S*)-HPMPA, cyclic(*S*)-HPMPA, (*S*)-HPMPDAP, cyclic(*S*)-HPMPDAP, 3-deaza-HPMPA, cyclic-3deaza-HPMPA, 7-deaza-HPMPA, cyclic-7-deaza-HPMPA, 8-aza-HPMPA, and cyclic-8-aza-HPMPA), HPMPO derivatives ((*R*)-HPMPO-DAPy and cyclic(*R*)-HPMPO-DAPy), and PMEO derivatives (PMEO-DAPy). Other DNA polymerase inhibitors were included for comparison: the natural tetracyclic diterpenoid aphidicolin, the pyrophosphate analogue PAA, and the nucleoside analogue AraC (cytosine arabinoside), considering that specific changes in the poxvirus DNA polymerase have been previously associated with resistance or hypersensitivity to these compounds [34,43]. The drug-susceptibility profiles of the cHPMPC-R and HPMPA-R clones were in line with our previously reported data derived from a recombinant VACV bearing the A314T DNA pol substitution [37]. The cHPMPA-R clones with the A353V + S851Y had a different drug-susceptibility profile compared to viral clones having the single S851Y change ([41] and Figure 1A). The A353V + S851Y changes led to higher levels of resistance to the HPMP derivatives and to HPMPO-DAPy, and to lower hypersensitivity to PMEO-DAPy and PAA than the single S851Y mutant. Consequently, the novel A353V change can be ascribed to altered drug susceptibility. Ala-353 maps to the 3′-5′ exonuclease domain of the DNA polymerase where several changes linked to altered drug susceptibility occur.

#### 3.1.2. CPXV Resistance to HPMPC, HPMPDAP, and HPMPO-DAPy

**Figure 2:**
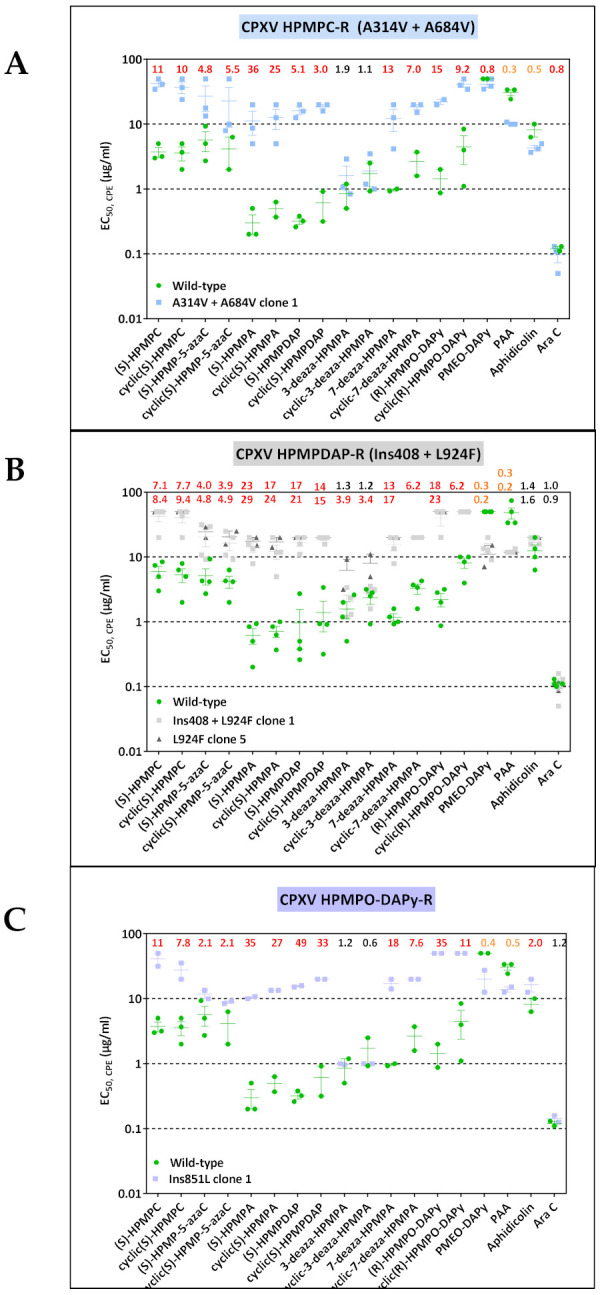
Drug-susceptibility profile of HPMPC-R (**A**), HPMPDAP-R (**B**), and HPMPO-DAPy-R (**C**) clones isolated from CPXV Brighton strain. Drug-resistance properties of different plaque-purified viral clones bearing the indicated DNA pol mutations as determined using a CPE reduction assay with HEL fibroblasts. The effects of different drugs on viruses encoding the indicated mutations were determined by calculating the EC_50_ values for the parental wild-type strain and clones bearing the specific mutations. At least two independent experiments were performed for each test compound. Horizontal lines for each drug and mutant indicate the mean values ± standard deviation. The fold resistance (ratio of the EC_50_ for the mutant virus to the EC_50_ for the wild-type CPXV strain) is marked at the top of the graph. CPXV viral clones showing a ≥2-fold increase (red, bold) were considered drug resistant (R) and those with a ≤0.5-fold decrease (orange, bold) drug hypersensitive (hs). For CPXV HPMPDAP-R, the fold resistance for the Ins408 + L924F mutant is indicated in the upper part of the figure line, while for the L924F mutant these numbers are shown underneath the previous ones.

Three ANPs, i.e., HPMPC, HPMPDAP, and HPMPO-DAPy, were used to characterize CPXV DNA pol mutants (Figure 2 and Table 2). Six viral clones were isolated following growth of the CPXV Brighton strain in the presence of HPMPC. All clones acquired the mutations A314V in combination with the A684V, two positions known to be associated with drug resistance [37,38,39,40]. Phenotyping of one of the CPXV HPMPC-R clones indicated that the combination of the A314V + A684V led to resistance to HPMP derivatives (except for the 3-deaza compounds), and HPMPO-DAPy compounds and hypersensitivity to PAA and aphidicolin and no changes in susceptibility to PMEO-DAPy and AraC.

All CPXV clones isolated under pressure of HPMPDAP harbored the novel DNA pol mutation L924F, with three out of eight clones presenting in addition an insertion of nine amino acids (KDIICKVIH) at position 408. The L924F change could be assigned to drug-resistance as evidenced by an increase in the EC_50_ values for all HPMP compounds except for the 3-deaza-analogues. Interestingly, the acquisition of the 408 insertion in the backbone of the L924F DNA pol mutant virus resulted in resistance to the 3-deaza-HPMPA drugs, while the hypersensitivity to PMEO-DAPy and PAA was retained. The L924F w/o the 408 insertion remained sensitive to AraC and aphidicolin.

CPXV clones arising under HPMPO-DAPy pressure bore an insertion of a leucine at position 851 (Ins851L), with the S851Y change being a known drug resistance mutation [41,42]. Phenotyping of the DNA pol mutant Ins851L showed that this insertion could be linked to resistance to all HPMP drugs (except for 3-deaza compounds) and to aphidicolin, as well as to hypersensitivity to PMEO-DAPy and PAA without changes in sensitivity to AraC.

#### 3.1.3. Genotypic Characterization of VACV DNA Pol Mutants Arising Following Four Independent Selection Procedures with HPMPC, HPMPDAP, and HPMPO-DAPy

As the vaccine strain Lederle contains viruses with different DNA polymerase natural genetic polymorphisms, we selected four plaque-purified viral clones, denoted VV1, VV2, VV8, and VV11, for drug-resistance selection under pressure of HPMPC, HPMPDAP, and HPMPO-DAPy. Clones VV1 and VV2 have at position 936–938 the sequence NΔG, while clones VV8 and VV11 have the amino acids ANV.

The VACV VV1, VV2, VV8, and VV11 clones were grown in increasing concentrations of three ANPs (HPMPC, HPMPDAP, HPMPO-DAPy). After 35–40 passages in increasing drug concentrations, viral clones were isolated and complete genotyping of the viral DNA polymerase was carried out for one viral clone isolated from each independent drug-resistance selection procedure (Table 3). The drug-resistant clones derived from VV1, VV2, VV8, and VV11 harbored at least one mutation previously described as linked to drug resistance (highlighted in dark blue boxes). The A314V/T, A613T, A684V, T688A and S851Y amino acid substitutions have been earlier identified in different poxviruses selected for resistance against HPMPC [37,38,39,40,42] or HPMPDAP [41]. In this study, the A314V/T change in the 3′-5′ exonuclease domain was found in combination with a mutation in the polymerase domain, with exception of the VV11 HPMPO-DAPy-R that only harbors the A314V change. The A314V/T was detected mostly in combination with A684V except for the VV1 HPMPDAP-R mutant virus (A314V + T688A).

The novel M313I DNA pol change in the 3′-5′ exonuclease domain was identified in three independent selection procedures (VV2 HPMPC-R, VV2 HPMPO-DAPy-R, and VV8 HPMPO-DAPy) in combination with the A684V. These data suggested that the M313I, which is next to the known A314T/V ANP-resistant mutation, may also be involved in altered drug susceptibility.

As well as M313I, several changes in the VACV DNA pol of unknown significance (Table 3, yellow cells) were identified following selection with HPMPC, HPMPDAP, and HPMPO-DAPy. Some of these substitutions are expected to be associated with changes in drug susceptibility because they map to conserved regions of the viral enzyme, A632T (region VI), and M656I, A677T, and L696S (region III).

### 3.2. Chronological Analysis of Emergence of DNA pol VACV Mutants Resistant to HPMPC, HPMPDAP, and HPMPO-DAPy

Because most of the viruses emerging after 40 passages under increasing concentrations of HPMPC, HPMPDAP, and HPMPO-DAPy harbored different mutations in the viral DNA polymerase, we investigated the sequence of appearance of the mutations in the various selection procedures. For this purpose, various plaque-purified viruses were isolated at different passages of the selection procedure and investigated for the presence of DNA pol changes.

**Figure 3:**
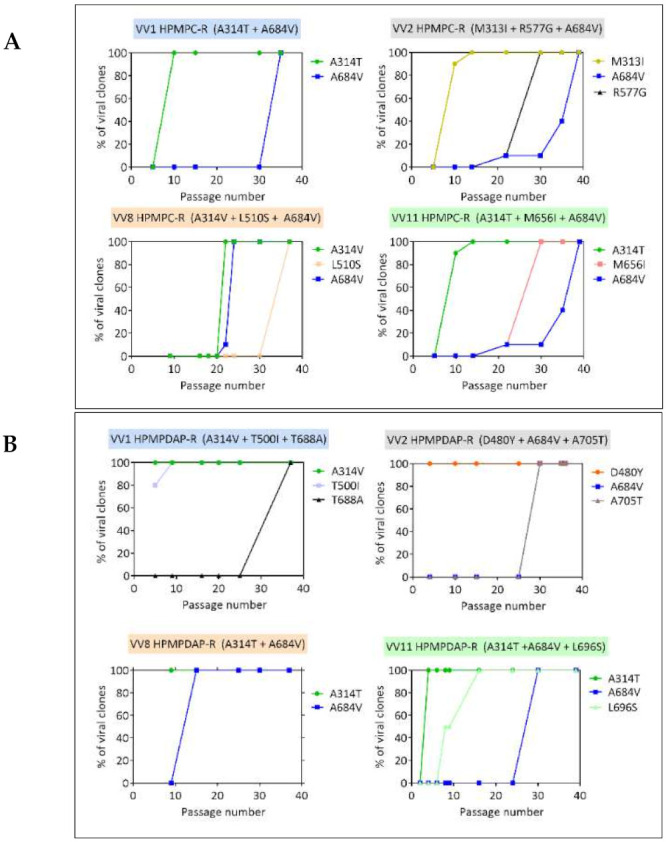

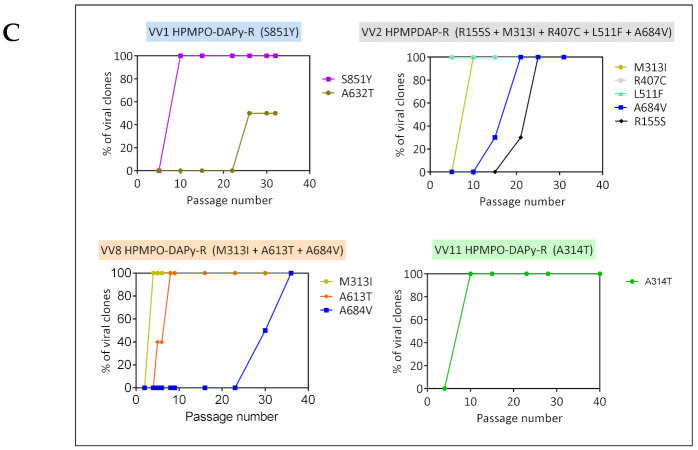
Chronological detection of DNA pol mutants arising at different passages under pressure of HPMPC (**A**), HPMPDAP (**B**), and HPMPO-DAPy (**C**). Viral clones were isolated at different passages during the selection procedure and genotyping of the E9L (DNA pol) gene was carried out. The percentage of clones harboring a specific mutation is shown for the different passages.

Mutations in the 3′-5′ exonuclease domain (M313I, A314T/V) emerged relatively fast and before the appearance of the A684V or T688A changes mapping to the polymerase domain (catalytic subunit) (Figure 3). Like the M313I and A314T/V, the D480Y, T500I, L511F, L696S, and S851Y DNA pol changes could be detected at early passages of the selection procedure. In contrast, the L510S, R577G, and M656I could be identified at later passages, like the A684V and T688A mutations.

### 3.3. Phenotypic Characterization of VACV Viral Clones Selected under Pressure of HPMPC, HPMPDAP, and HPMPO-DAPy

To unravel the impact of novel mutations in the VACV DNA polymerase identified following selection with HPMPC, HPMPDAP, and HPMPO-DAPy, the susceptibility profile of VACV bearing specific DNA pol mutations was performed by CPE reduction assay in HEL fibroblasts. For this purpose, plaque-purified viral clones recovered at different passages during the drug-selection procedures were used.

#### 3.3.1. VACV Clones Emerging under HPMPC Pressure

VV1 HPMPC-R clones presented the known A314T + A684V changes in addition to the novel R97H substitution (Figure S3A). The drug-susceptibility profile of this triple mutant (Figure S3B) was comparable to that found for the A314T + A684V recombinant virus [37], suggesting the association of the R97H change, not located in the active site of the viral enzyme, with a naturally occurring polymorphism.

**Figure 4:**
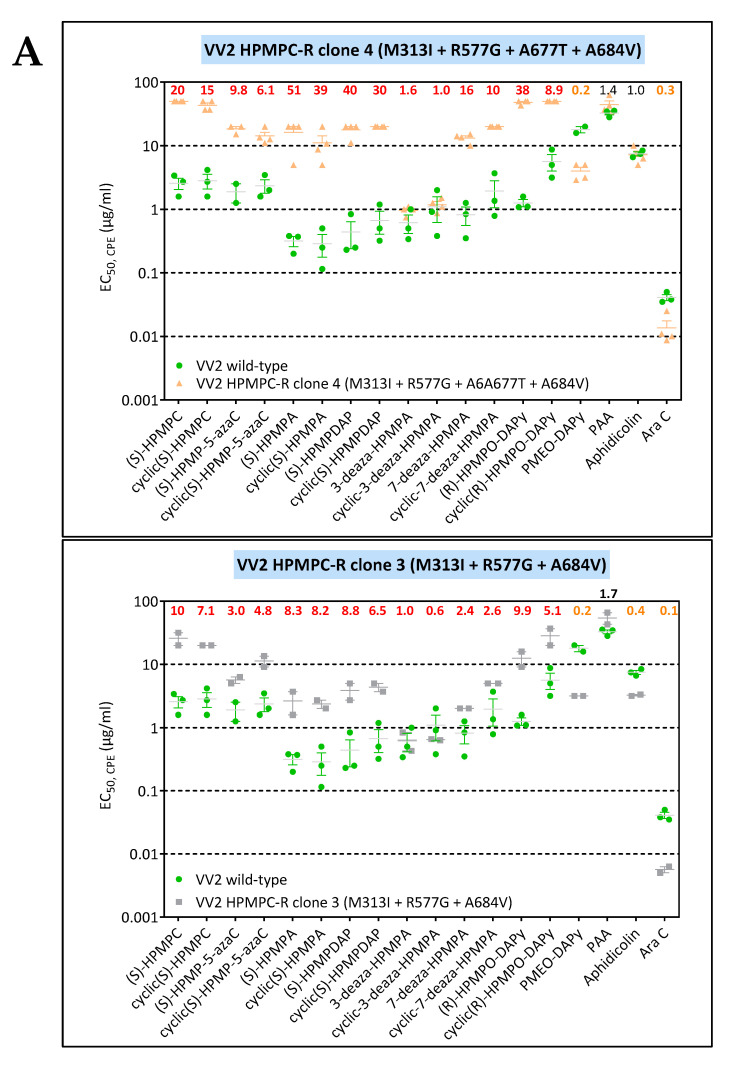

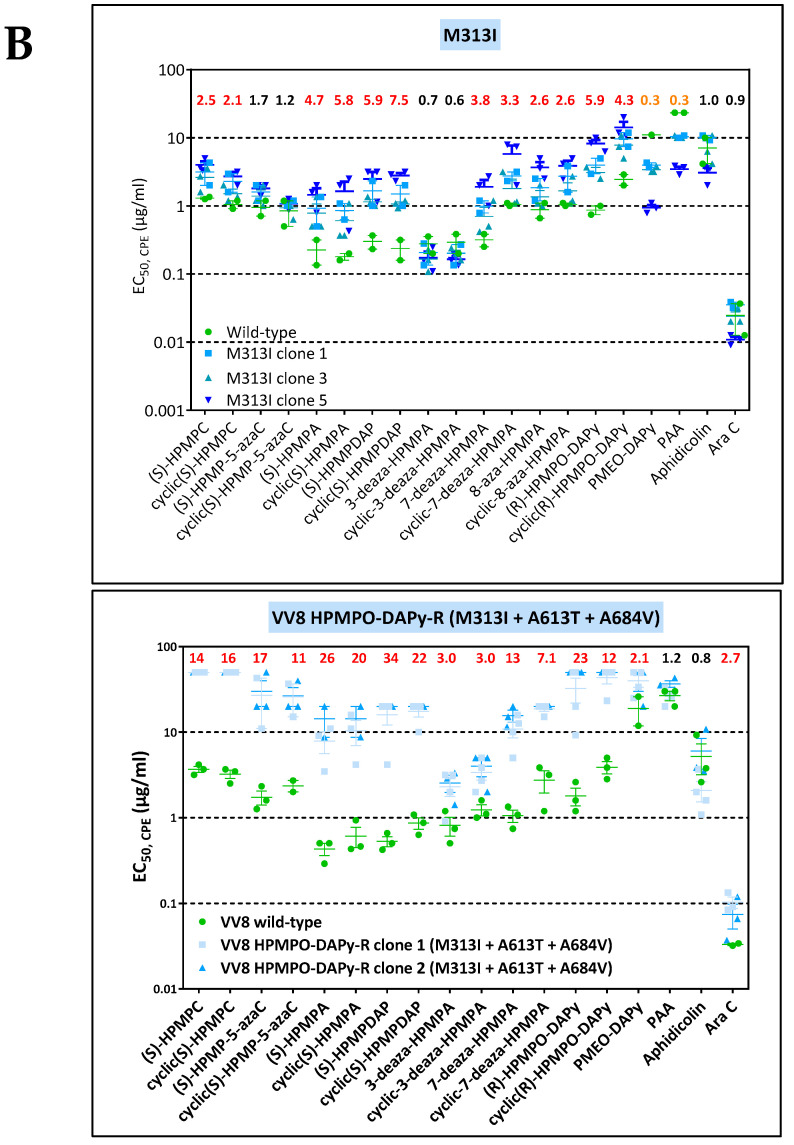

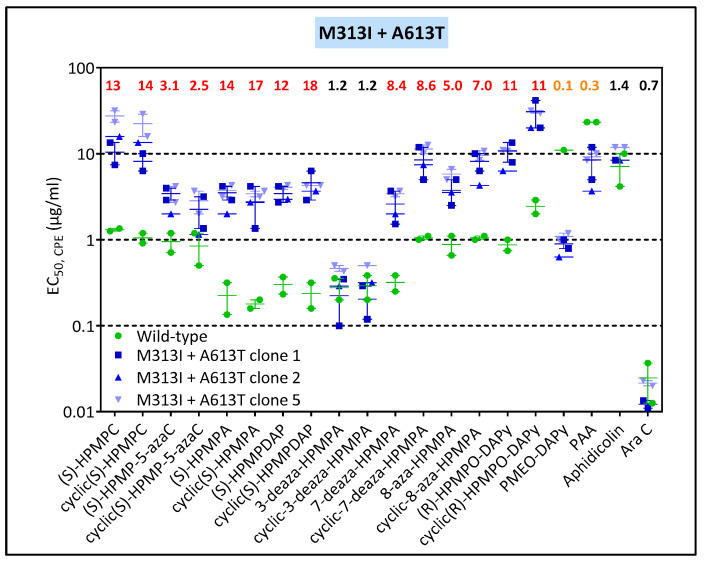
(**A**) Drug-susceptibility profile of selected VV2 viral clones bearing the M313I + R577G + A684V w/o the A677T change. All clones harbored the W8C substitution, which is expected to be linked to a natural occurring genetic polymorphism since it maps to the beginning of the N-terminal structural domain where drug-resistance mutations have not been described. Drug-resistance properties of the two viral clones were determined using a CPE reduction assay with HEL fibroblasts. (**B**) Drug-susceptibility profile of selected VV8 HPMPO-DAPy-R viral clones to evaluate the impact of the M313I, R407C, L511F, and A613T DNA polymerase substitutions. The source of the viral clones was as follows: M313I (VV8 HPMPO-DAPy #4), M313I + A613T (VV8 HPMPO-DAPy #9), and M313I + A613T+ A684V (VV8 HPMPO-DAPy-R #36). Drug-resistance properties of the different types of viral clones were determined using a CPE reduction assay with HEL fibroblasts. The effects of different drugs on viruses encoding the indicated mutations were determined by calculating the EC_50_ values for the parental wild-type strain and clones bearing the specific mutations. At least two independent experiments were performed for each test compound. Horizontal lines for each drug and mutant viral clones indicate the mean values ± standard deviation. The fold resistance (ratio of the EC_50_ for the mutant viruses to the EC_50_ for the corresponding wild-type virus is marked at the top of the graph. VACV viral clones showing a ≥2-fold increase (red, bold) were considered drug resistant (R) and those with a ≤0.5-fold decrease (orange, bold) drug hypersensitive (hs).

VV2 HPMPC-R clone 2 harbored the W8C, M313I, R577G, A677T, and A684V substitutions, being all novel changes except for A684V. When analyzing the various viral clones recovered from this selection procedure, some of them lacked the A677T change (Table S2). A viral clone bearing the W8C + M313I + R577G + A684V substitutions showed resistance to HPMP (except 3-deaza compounds) and HPMPO-DAPy derivatives and hypersensitivity to PMEO-DAPy, aphidicolin, and AraC (Figure 4A). The additional presence of the A677T resulted in higher levels of resistance to HPMP and HPMPO-DAPy drugs, indicating that the A677T influences drug susceptibility. Except for the W8C substitution, the M313I, R577G, A677T, and A684V map to the active site of the enzyme, but from our data it is not possible to infer the contribution to drug resistance of each mutation. Since the W8C is located at the beginning of the N-terminal structural domain (amino acids 1–157) in a region where drug-resistance mutations are not described, it can be assumed that this change most likely is related to a naturally occurring genetic polymorphism.

**Figure 5:**
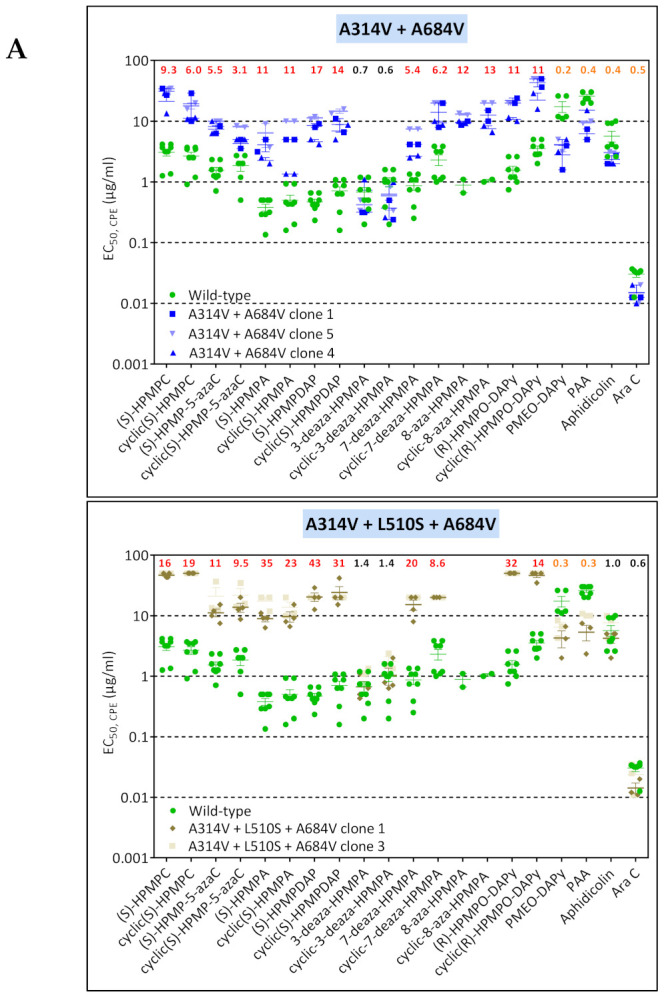

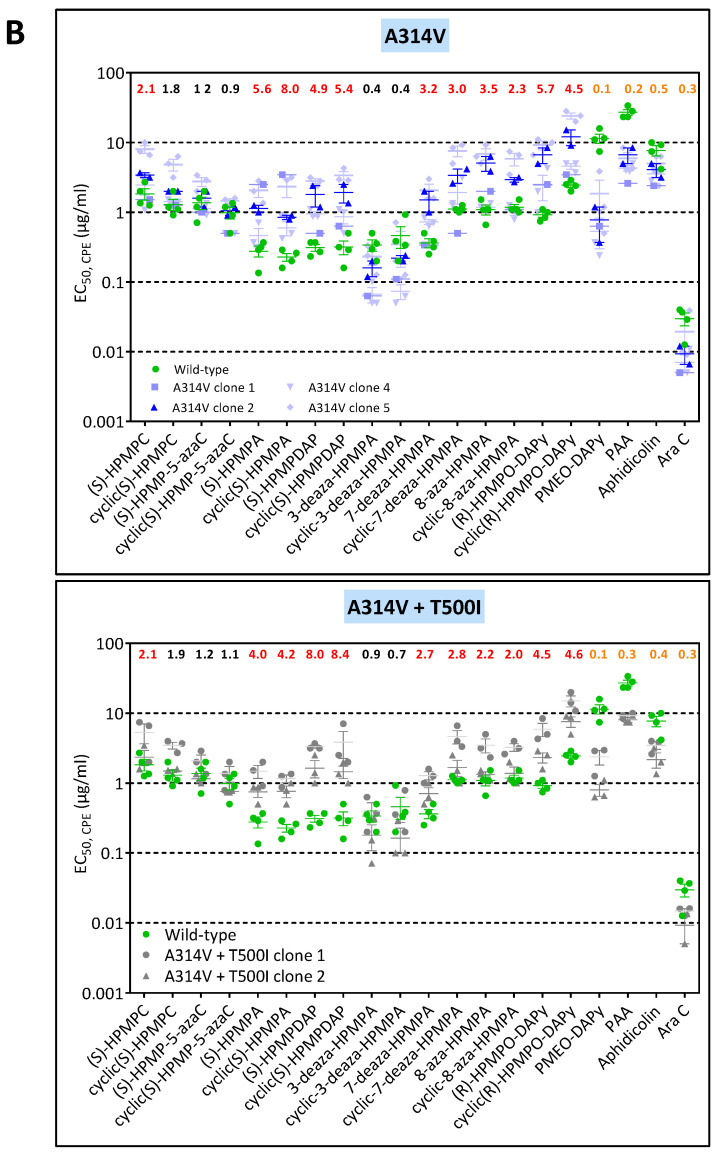

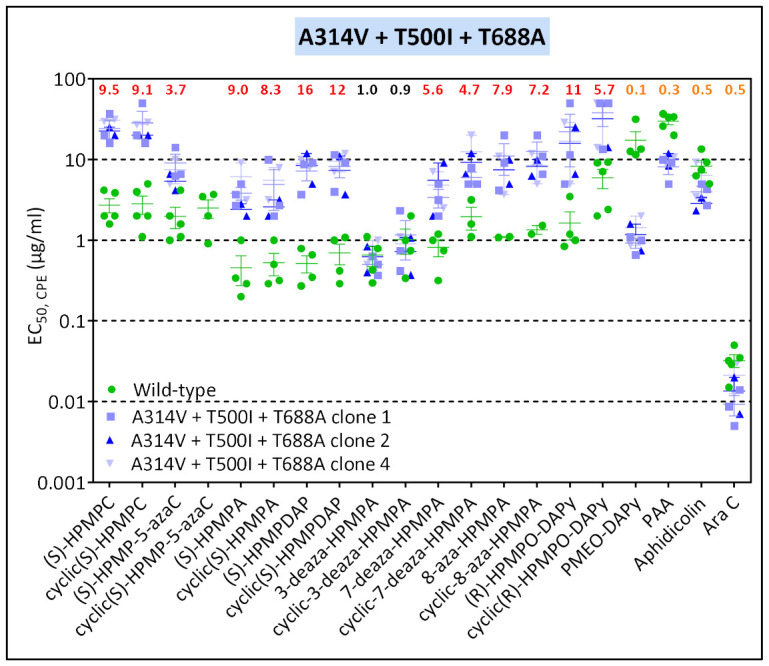
(**A**) Drug-susceptibility profile of selected VV8 viral clones to evaluate the impact of the A314V, L510S, and A684V DNA polymerase substitutions. The source of the viral clones was as follows: A314V + A684V (VV8 HPMPC-R #30) and the A314V + L510S + A684V (VV8 HPMPC-R #37). (**B**) Drug-susceptibility profile of selected viral clones to evaluate the impact of the A314V, T500I, and T688A DNA polymerase substitutions. The source of the viral clones was as follows: A314V (VV8 HPMPC-R #22), A314V + T500I (VV1 HPMPDAP-R #9), and A314V + T500I + T688A (VV1 HPMPDAP-R #37). Drug-resistance properties of the different types of viral clones were determined using a CPE reduction assay with HEL fibroblasts. The effects of different drugs on viruses encoding the indicated mutations were determined by calculating the EC_50_ values for the parental wild-type virus and clones bearing the specific mutations. At least two independent experiments were performed for each test compound. Horizontal lines for each drug and mutant viral clones indicate the mean values ± standard deviation. The fold resistance (ratio of the EC50 for the mutant viruses to the EC50 for the corresponding wild-type virus clone is marked at the top of the graph. VACV viral clones showing a ≥2-fold increase (red, bold) were considered drug resistant (R) and those with a ≤0.5-fold decrease (orange, bold) drug hypersensitive (hs).

The known drug-resistant mutations A314V and A684V were found together with the novel L510S change in the VV8 HPMPC-R clone 1. Since the L510S was acquired after the emergence of the A314V + A684V change, clones isolated from intermediate passages were analyzed phenotypically to investigate the impact of this mutation. Compared to the single A314V substitution, the combination of the A314V with the A684V resulted in higher levels of resistance to the HPMP and HPMPO compounds while preserving the hypersensitivity to PMEO-DAPy, PAA, Aphidicolin, and AraC but not to the 3-deaza- HPMPA drugs. Adding the L510S to a virus bearing the A314V/A684V increased the resistance levels to HPMP and HPMPO compounds and hindered hypersensitivity to Aphidicolin and AraC (Figure 5A), pointing to an impact of the L510S change in drug susceptibility.

The VV1 HPMPDAP-R virus rapidly acquired the A314V and T500I changes. After five passages, the A314V and T500I substitutions were detected in 100% and 80% of the clones, respectively. The A314V VV1 clones displayed low levels of resistance to several ANPs but marked hypersensitivity to the 3-deaza derivatives, PMEO-DAPy, PAA, aphidicolin, and AraC (Figure 5B). Addition of the T500I to the A314V did not result in substantial changes in the level of resistance nor in the pattern of the drug-susceptibility profile. Acquisition of the T688A substitution in the backbone of the A314V + T500I led to higher levels of resistance to HPMP (except for the 3-deaza derivatives) and HPMPO derivatives, and maintenance of the hypersensitivity to PMEO-DAPy, PAA, aphidicolin, and AraC. (Figure 5B).

**Figure 6:**
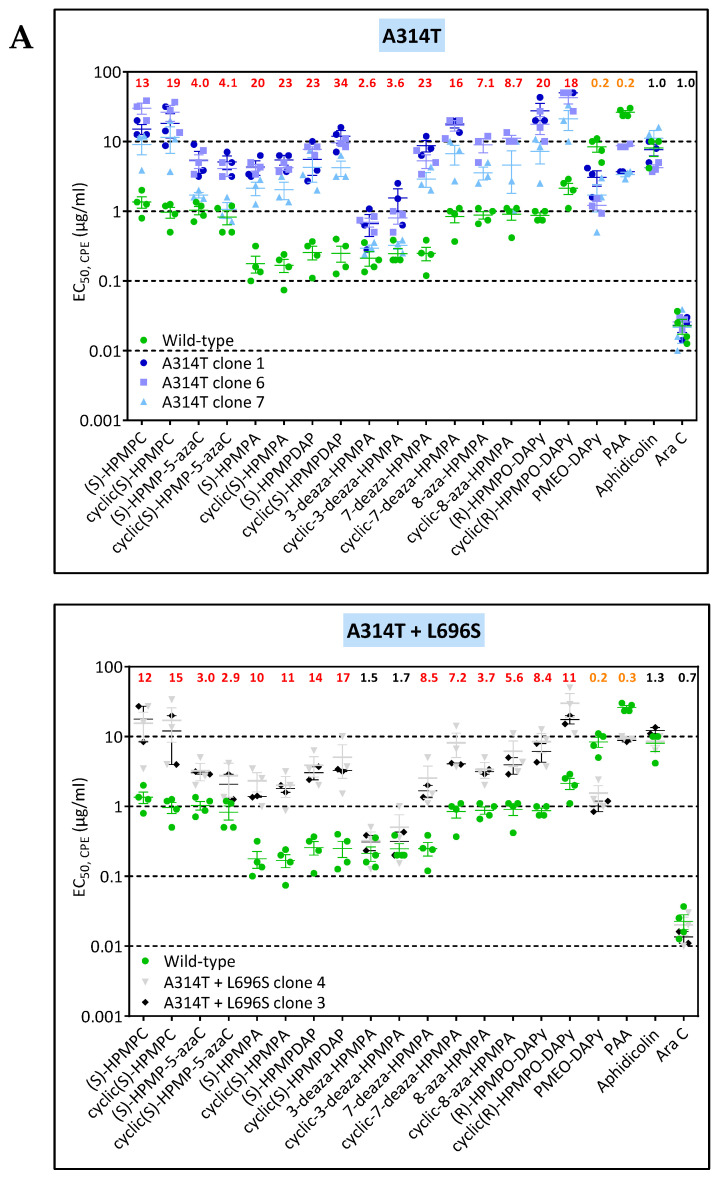

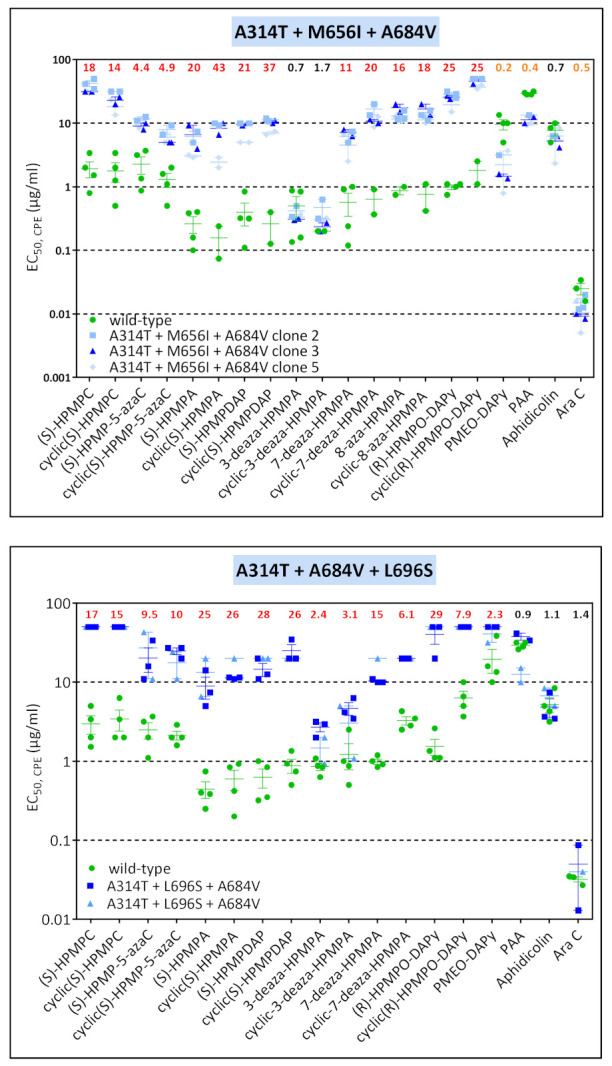

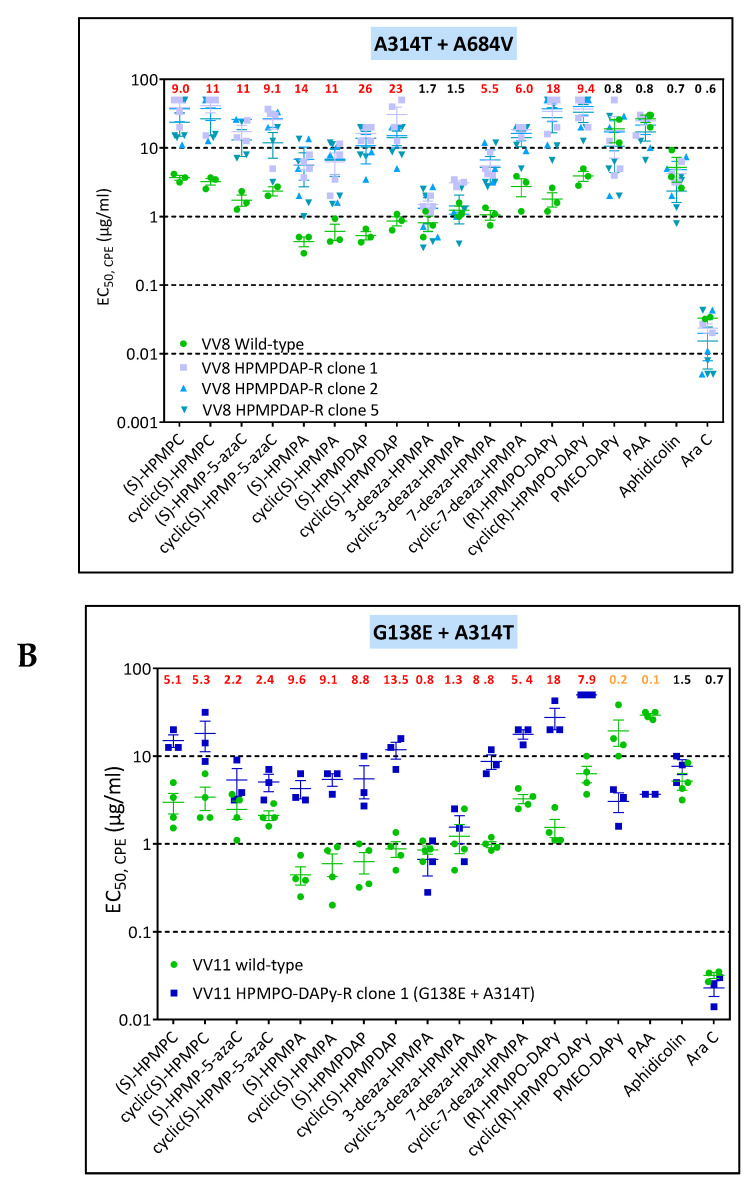
(**A**) Drug-susceptibility profile of selected viral clones to evaluate the impact of the A314T, M656I, A684V, and L696S DNA polymerase substitutions. The source of the viral clones was as follows: A314T (VV8 HPMPDAP-R #8), A314T + A684V (VV8 HPMPC-R #30), A314T + L696S (VV11 #16), A314T + M656I + A684V (VV11 HPMPC-R #40), and A314T + A684V + L696S (VV11 HPMPDAP-R #39). (**B**) Drug-susceptibility profile of the selected VV11 HPMPO-DAPy-R viral clone bearing the G138E + A314T mutations. Drug-resistance properties of the different types of viral clones were determined using a CPE reduction assay with HEL fibroblasts. The effects of different drugs on viruses encoding the indicated mutations were determined by calculating the EC_50_ values for the parental wild-type strain and clones bearing the specific mutations. At least two independent experiments were performed for each test compound. Horizontal lines for each drug and mutant viral clones indicate the mean values ± standard deviation. The fold resistance (ratio of the EC_50_ for the mutant viruses to the EC_50_ for the corresponding wild-type virus is marked at the top of the graph. VACV viral clones showing a ≥2-fold increase (red, bold) were considered drug resistant (R) and those with a ≤0.5-fold decrease (orange, bold) drug hypersensitive (hs).

VV11 HPMPC-R clone 1 had the previously reported drug-resistance changes A314T and A684V in combination with the novel changes L90S, R234L, M656I, and S898T. However, not all clones harbored the mutations at positions 90, 234, and 898 (Table S3). When comparing the drug-susceptibility profile of viral clones with the A314T+ M656I + A684V with or without the L90S, R234L, and S898T changes, no substantial differences were found, suggesting that the L90S, R234L, and S898T substitutions are related to inter-strain variability. In contrast, the M656I change had an impact on drug susceptibility as the A314T + M656I + A684V mutant showed clear hypersensitivity to PMEO-DAPy and PAA in contrast to the A314T + A684V mutant (Figure 6A).

#### 3.3.2. VACV Clones Emerging under HPMPDAP Pressure

The VV1 HPMPDAP-R virus rapidly acquired the A314V and T500I changes. After five passages, the A314V and T500I substitutions were detected in 100% and 80% of the clones, respectively. The A314V VV1 clones displayed low levels of resistance to several ANPs but marked hypersensitivity to the 3-deaza derivatives, PMEO-DAPy, PAA, Aphidicolin, and AraC) (Figure 5B). Addition of the T500I to the A314V did not result in substantial changes in the level of resistance nor in the pattern of drug-susceptibility profile. Acquisition of the T688A substitution in the backbone of the A314V + T500I led to higher levels of resistance to HPMP (except for the 3-deaza derivatives) and HPMPO derivatives, and maintenance of the hypersensitivity to PMEO-DAPy, PAA, aphidicolin, and AraC (Figure 5B).

**Figure 7:**
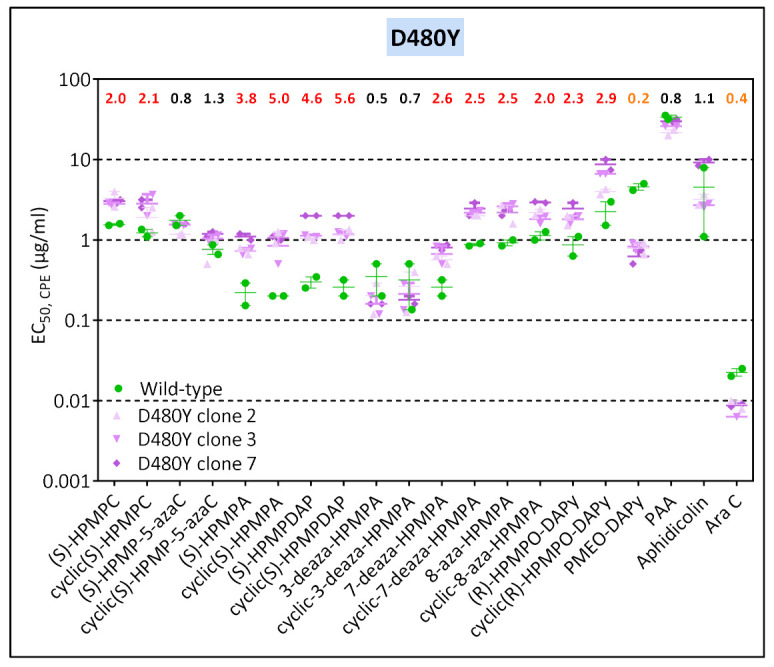

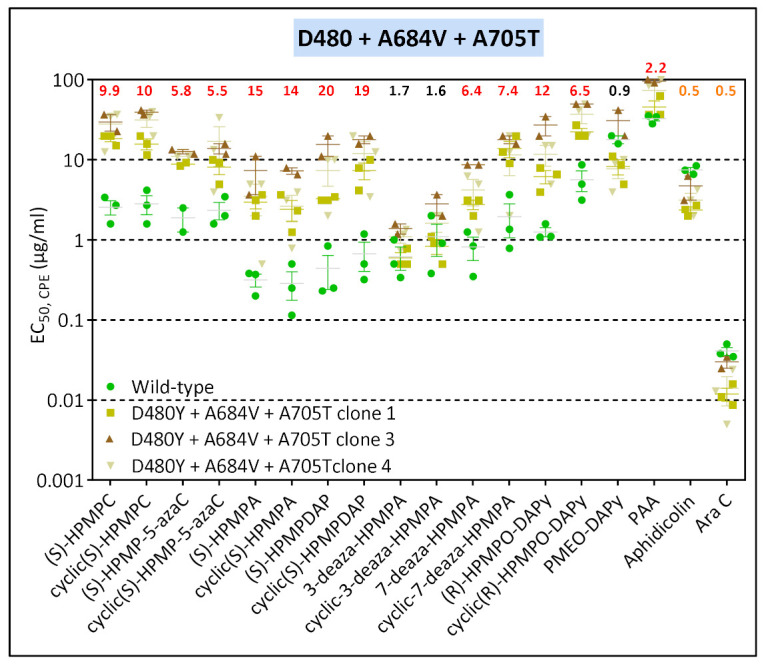
Drug-susceptibility profile of selected VV2 HPMPDAP-R viral clones to evaluate the impact of the D480Y, A684V, and A705T DNA polymerase substitutions. The source of the viral clones was as follows: D480Y (VV2 HPMPDAP-R # 4), and D480Y + A684V + A705T (VV2 HPMPDAP-R #36). Drug-resistance properties of the different types of viral clones were determined using a CPE reduction assay with HEL fibroblasts. The effects of different drugs on viruses encoding the indicated mutations were determined by calculating the EC_50_ values for the parental wild-type strain and clones bearing the specific mutations. At least two independent experiments were performed for each test compound. Horizontal lines for each drug and mutant viral clones indicate the mean values ± standard deviation. The fold resistance (ratio of the EC_50_ for the mutant viruses to the EC_50_ for the corresponding wild-type virus is marked at the top of the graph. VACV viral clones showing a ≥2-fold increase (red, bold) were considered drug resistant (R) and those with a ≤0.5-fold decrease (orange, bold) drug hypersensitive (hs).

Mutants recovered from the VV2 HPMPDAP selection procedure acquired rapidly the D408 substitution, with 100% of the clones having this change only after five passages (Figure 2). In contrast, the A684V and A705T mutations were detected after 30 passages and appeared to occur simultaneously. Viral clones bearing the D480Y change showed low levels of resistance to the HPMP compounds (except for HPMP-5-azaC, 3-deaza-HPMPA, and their cyclic forms, and hypersensitivity to PMEO-DAPy and AraC (Figure 7). The combination of the D480Y together with the A684V and A705T mutations resulted in increased resistance to HPMP derivatives, except for the 3-deaza compounds, and loss of hypersensitivity to PMEO-DAPy and PAA together with hypersensitivity to aphidicolin and AraC. These data indicated that the D480Y change could be ascribed to drug resistance, while the contribution of the A705T substitution cannot be undoubtable determined due to its association with the A684V.

The clones derived from VV8 under pressure with HPMPDAP bore the known drug-resistance mutations A314T + A684V, whereas those arising from VV11 had in addition the L696V change. In both cases, the A314T change emerged first, followed by the L696V and then the A684V in the case of VV11 (Figure 2). The A314T and A314V had a different effect on the drug-susceptibility/resistance profile. The A314T led to higher levels of resistance to the HPMP and HPMPO-DAPy compounds than the A314V and hypersensitivity to PMEO-DAPy and PAA but not to aphidicolin and to AraC, in contrast to the A314V that showed hypersensitivity to these four DNA pol inhibitors. When the A314T was combined with the A684V, the hypersensitivity to PMEO-DAPy and PAA was reverted but not when A314T was in combination with the L696S (Figure 6B). When the three changes A314T + A684V + L696S were present, resistance to all classes of ANPs was observed, while sensitivity to PAA, aphidicolin, and AraC were not changed compared to the wild-type virus. The loss of hypersensitivity to both PMEO-DAPy and PAA following acquisition of the A684V change by the A314T mutant virus is in line with our previous data obtained with recombinant viruses bearing these specific amino acid changes in the DNA polymerase (2).

#### 3.3.3. VACV Clones Emerging under HPMPO-DAPy Pressure

VV1 HPMPO-DAPy-R viruses harbored the novel A632T mutation and the known drug-resistant S851Y mutation, with the S851Y emerging first. When comparing the sensitivity of the S851Y mutant (Figure 1A and data derived from a recombinant virus (41)) and data obtained with the A632T + S851Y mutant, it can be concluded that the A632T increases the level of resistance to HPMP and HPMPO-DAPy derivatives, while maintaining the hypersensitivity to PAA and PMEO-DAPy (Figure 1B).

**Figure 8:**
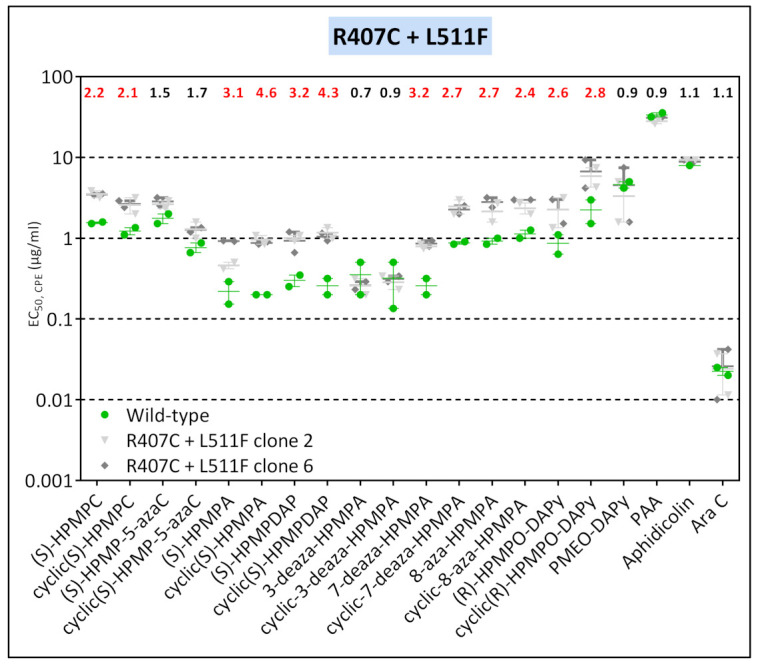

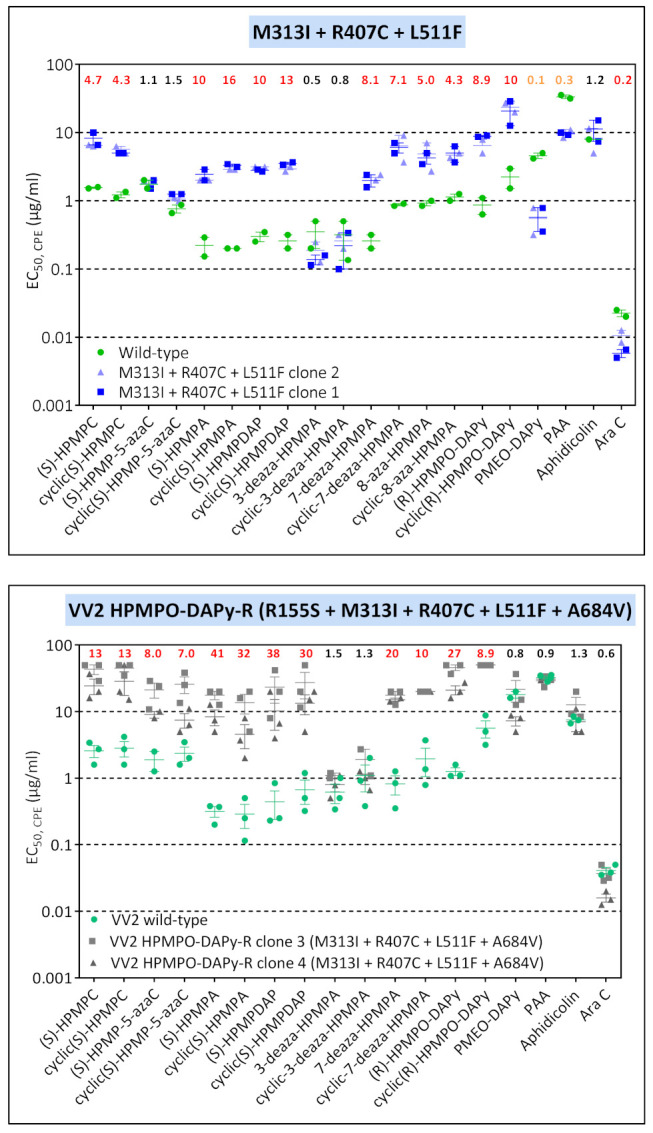
Drug-susceptibility profile of selected VV2 HPMPO-DAPy-R viral clones to evaluate the impact of the M313I, R407C, L511F, and A613T DNA polymerase substitutions. The source of the viral clones was as follows: R407C + L511F (VV2 HPMPO-DAPy-R #5), M313I + R407C + L511F (VV2 HPMPO-DAPy-R #10), and R155S + M313I + R407C + L511F + A684V (VV2 HPMPO-DAPy-R #31). Drug-resistance properties of the different types of viral clones were determined using a CPE reduction assay with HEL fibroblasts. The effects of different drugs on viruses encoding the indicated mutations were determined by calculating the EC50 values for the parental wild-type strain and clones bearing the specific mutations. At least two independent experiments were performed for each test compound. Horizontal lines for each drug and mutant viral clones indicate the mean values ± standard deviation. The fold resistance (ratio of the EC50 for the mutant viruses to the EC50 for the wild-type VV2 is marked at the top of the graph. VACV viral clones showing a ≥2-fold increase (red, bold) were considered drug resistant (R) and those with a ≤0.5-fold decrease (orange, bold) drug hypersensitive (hs).

VV2 mutant viruses arising under pressure with HPMPO-DAPy acquired several mutations, with the novel changes R407C and L511F detected already after five passages, followed by the emergence of M313I and A684V changes, found, respectively, at passages 10 and 15 (Figure 2). The R407 + L511F mutant clones displayed low levels of resistance to HPMP (except for 3-deaza-HPMPA and HPMP-5-aza compounds) and to HPMPO-DAPy, but the acquisition of the M313I in a backbone of the R407C + L511F virus resulted in increased resistance to the same ANPs and marked hypersensitivity to PMEO-DAPy, PAA, and AraC but not to aphidicolin (Figure 8). The addition of the R155S + A684V changes to the R407C + L511F + M313I virus led to even higher levels of resistance to HPMP (except for 3-deza-HPMPA compounds) and HPMPO-DAPy derivatives and the loss of hypersensitivity to PMEO-DAPy, PAA, and AraC.

The M313I substitution was also found following selection under pressure of HPMPO-DAPy starting from VV8 at very early stages of the selection procedure (after four passages). The M313I virus acquired quite fast the A613T change (after five passages), whereas the A684V change arose at passage 30 (Figure 2). Clones bearing the M313I mutation showed a low level of resistance to HPMP (except 5-azaC and 3-deaza drugs) and HPMPO-DAPy compounds and hypersensitivity to PMEO-DAPy and PAA (Figure 4B). Addition of the A613T to the M313I did not change the drug-susceptibility/resistance profile but, indeed, increased the resistance levels. The virus bearing the triple combination M313I + A613T + A684V showed resistance to all ANPs and to AraC and was as sensitive as the wild-type to PAA and aphidicolin. The A613T has been previously described in MPXV in combination with the A314V, A684V, and T808M changes [38]. In the VV8 HPMPO-DAPy virus, A613T was detected together with M313I, a mutation also identified following two other independent processes of selection (i.e., VV2 HPMPC-R and VV2 HPMPO-DAPy).

When VV11 was grown in the presence of HPMPO-DAPy, the known A314T drug-resistance mutation could be detected after 10 passages under pressure with this ANP (Figure 2). After 40 passages, several clones were isolated and only 1 out of 10 clones had an additional new mutation (G138E). When the sensitivity of the G138E + A314T viral clone was compared with that of the A314T clones, it can be deduced that the addition of the G138E change did not modify the hypersensitivity to PMEO-DAPy and PAA but reduced the level of resistance to the HPMP derivatives (Figure 6A,B).

### 3.4. Characterization of Drug-Resistance VACV against PAA and PMEO-DAPy

Considering the cross-resistance or cross-hypersensitivity of PMEO-DAPy and PAA, we then performed a selection of mutant viruses using both drugs. The VV1, VV2, VV8, and VV11 VACV clones were grown in increasing concentrations of PAA or PMEO-DAPy. After approximately 40 passages, viral clones were isolated and one viral clone for each drug-resistance selection condition was characterized by analysis of the complete viral DNA polymerase (Table 4). Except for the VV2 clone 3 with the A684V change, all viruses emerging under PFA bore novel changes in the viral DNA polymerase. The F354L (VV1), 117Ins GISPD + E792D (VV8), and Y332H (VV11) changes are in the 3′-5′ exonuclease domain, where PAA-R mutations associated with an antimutator phenotype C356Y, G372D, and G380S, have been formerly reported [44]. Interestingly, under PFA, the A684V did not emerge in combination with DNA polymerase changes occurring in the 3′-5′ exonuclease domain.

**Figure 9:**
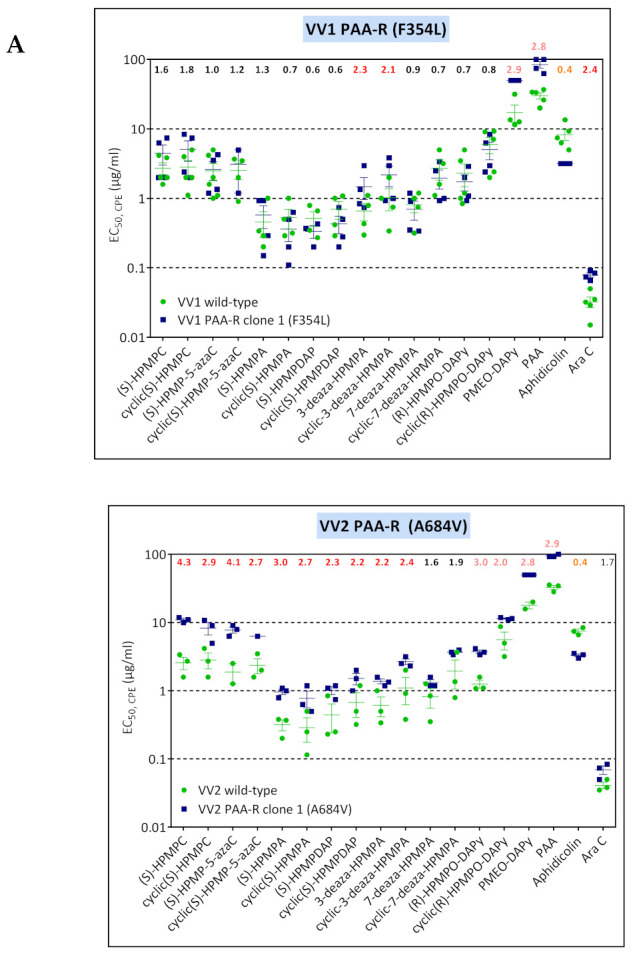

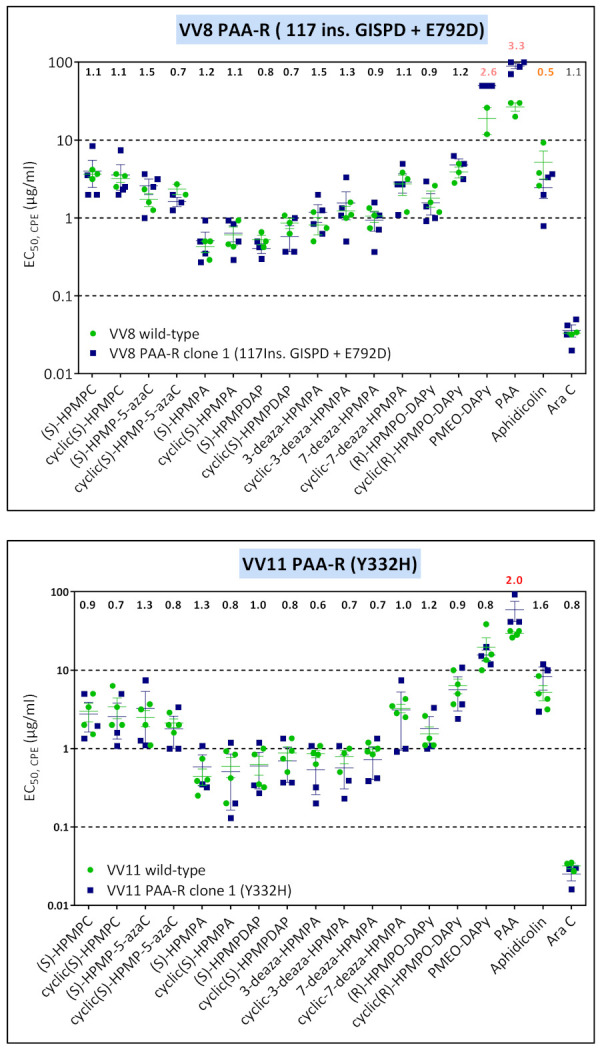

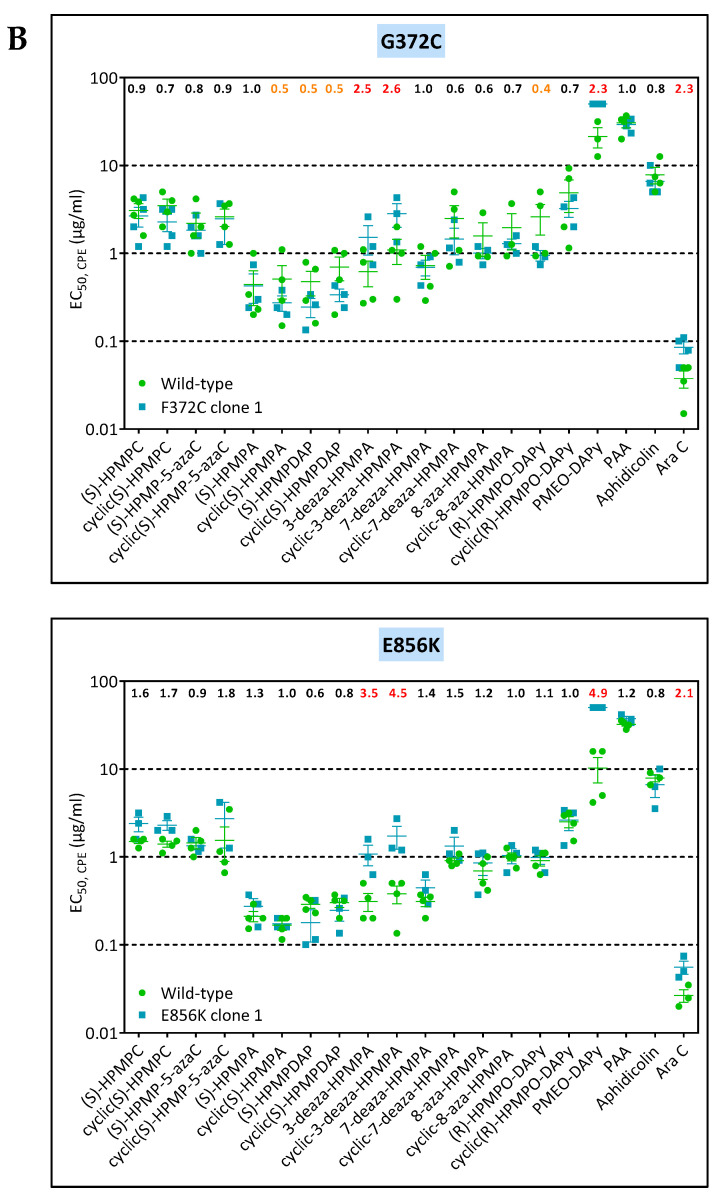
Drug-susceptibility profile of PAA-R viral clones (**A**) and PMEO-DAPy-R viral clones (**B**). Drug-resistance properties of the different viral clones were determined using a CPE reduction assay with HEL fibroblasts. The effects of different drugs on viruses encoding the indicated mutations were determined by calculating the EC_50_ values for the parental wild-type strain and clones bearing the specific mutations. At least two independent experiments were performed for each test compound. Horizontal lines for each drug and mutant viral clones indicate the mean values ± standard deviation. The fold resistance (ratio of the EC_50_ for the mutant viruses to the EC_50_ for the corresponding wild-type virus is marked at the top of the graph. VACV viral clones showing a ≥2-fold increase (red) were considered drug resistant (R) and those with a ≤0.5-fold decrease (orange) were considered drug hypersensitive (hs).

The four VACV PAA-R mutants isolated in the present study showed different patterns of drug susceptibility/resistance associated with specific changes in the viral DNA polymerase (Figure 9A). The A684V, found as a single change in the VV2 PAA-R virus, conferred low levels of resistance to all ANPs (except for 3-deaza-HPMPA derivatives) in addition to PAA and substantial aphidicolin hypersensitivity. Except for the Y332H mutant that showed a 2-fold resistance to PAA, cross-resistance between PAA and PMEO-DAPy and aphidicolin hypersensitivity was observed. Moreover, the F354L substitution was associated with low levels of resistance to 3-deaza compounds and AraC.

Two mutant viruses emerging under pressure with PMEO-DAPy were characterized geno- and phenotypically (Table 4 and Figure 9B). The G372C change in the 3′-5′- exonuclease domain (VV1) and the E856K in the polymerase domain (VV2) were identified. Interestingly, both mutants showed resistance to PMEO-DAPy, 3-deaza compounds, and AraC but not to PAA.

### 3.5. Topological Distribution of Drug-Ressitance Mutations in Poxvirus DNA Polymerase

Most of the known mutations linked to drug resistance, as well as those described here, clustered to either the exonuclease domain responsible for the proofreading activity of poxvirus DNA polymerases or to the domain responsible for the catalytic activity of the viral enzyme.

**Figure 10:**
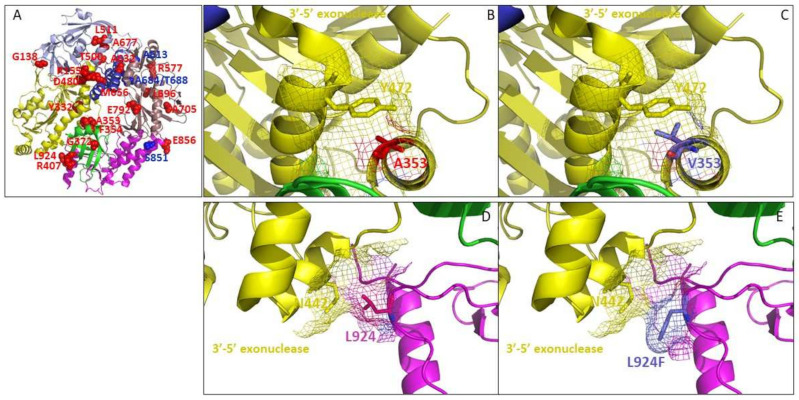
(**A**) Three-dimensional structure of VACV DNA polymerase (pdb code: 5N2E) harboring newly identified amino acid changes (represented as red spheres) and amino acid changes associated to drug resistance, described in the literature (as blue sphere). The polymerase and exonuclease functional domains are shown based on Tarbouriech et al.’s (45) characterization: finger (in blue), palm (in copper) and thumb (in magenta) domains catalyzing the polymerase activity, the 3′-5′ exonuclease domain (in yellow) associated to proofreading activity, and the NH2-terminal domain (in light blue). (**B**) Position 353 (in red) is located in the 3′-5′ exonuclease domain and (**C**) A353V change (in blue) might be involved in steric hindrance with residue Y472 (in yellow) (side chain volumes: Ala = 88.6 Å3 and Val = 140 Å3). Amino acid changes located in the exonuclease domain were described to be associated with drug resistance. (**D**,**E**) Interface of the 3′-5′ exonuclease (in yellow) and thumb (in magenta) domains showing the position of residue L924 (in red), F924 (in blue), and N442 (in yellow). The images were generated using PyMol Delano Software, version 0.99rc6.

The three-dimensional structure of the VACV DNA polymerase (E9 protein), characterized in 2017 by Tarbouriech and colleagues [45], was used to assess the effect of several amino acid changes identified in our study. Figure 10A shows the position of the amino acids that were found altered in the different drug-resistant clones described in our study. Using sphere representation of the atoms, and blue and red colors to discriminate between already characterized drug-resistance changes and newly identified variants, respectively, we observed that the amino acid changes are distributed in the different functional domains of the enzyme, affecting the polymerase and exonuclease functions.

#### 3.5.1. A353V and L924F May Affect the 3′-5′ Exonuclease Domain of Poxvirus E9 Protein

Residue A353, located in the 3′-5′ exonuclease domain, is found altered (A353V), together with S851Y, in drug-resistant VACV clones selected with cHPMPA (Figure 10B,C). It was described in the literature that S851Y alone affects the polymerase activity [41]. In addition, the susceptibility profile of VACV clones exhibiting the A353V change together with S851Y showed increased fold resistant change to HPMP analogues in comparison with VACV clones presenting the S851Y change alone. Taken together, we can hypothesize that A353V and S851Y may affect the 3′-5′ exonuclease and polymerase functions, respectively, result in increased fold resistance. Interestingly, the S851Y variation conferred hypersensitivity to 3-deaza-HPMPA, while A353V + S851Y together abolished the observed change in sensitivity to the drug.

Modification of the 3′-5′ exonuclease domain environment is also observed with the amino acid change L924F identified in HPMPDAP-R CPXV clones (CPXV and VACV DNA polymerases share 98.3% sequence identity). Thus, the three-dimensional structure of VACV DNApol can be used to analyze the effect of amino acid changes in CPXV E9 protein). Residue 924 of the thumb domain is in close vicinity with the asparagine residue at position 442 (Figure 10D,E). The change of a leucine to a phenylalanine resulted in the presence of a bulkier side chain (189.9 Å3 for Phe instead of 166.7 Å3 for Leu) that could impact the exonuclease domain. The M313I effects on 3′-5′ exonuclease function could not be analyzed because the region 307-HKGVGGM-313 is missing from the published structure. However, since the well-characterized A314V change has been associated to drug resistance, we can hypothesize that M313I could also be responsible for HPMPC resistance. R155S and D480Y amino acid changes are at the edge (aa155) or within (aa480) the exonuclease domain and may affect its function (Figure 10A). Interestingly, D480Y does not confer resistance to the 5azaC- and 3-deaza-analogues, suggesting that drug resistance through this specific mutation might be dependent on base conformation.

#### 3.5.2. The A632T, M656I, and A677T Might Alter the Positioning of the Fingers Domain to Avoid Incorporation of ANPs

**Figure 11:**
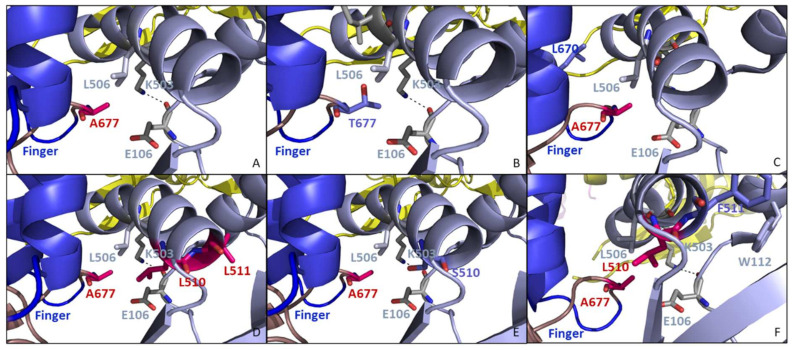
Structure of the interface finger (in blue)/NH2-terminal (in light blue) domains. (**A**,**B**) A677 (in red) is facing residues E106, K503, and L506 (in grey) at the interface between finger (in blue) and NH2-terminal (in light blue) domains. (**C**) Change at position 670 has been associated with aphidicolin resistance. (**D**,**E**) Residue L510 was found altered in our study through change L510S selected under the pressure with HPMPC, (**F**) while at position 511, a phenylalanine residue was present in the clones selected under pressure with HPMPO-DAPY. The images were generated using PyMol Delano Software, version 0.99rc6.

The residue 677 is located at the extremity of one of the two α helices that compose the finger domain. It is surrounded by residues E106, K50,3 and L506 that belong to the N-terminal domain of the DNA pol (Figure 11A). The A677T change induced an increase in the volume of the side chain (Ala = 88.6 Å3 and Thr = 116.1 Å3) (Figure 11B). This change might indirectly affect the polymerase function since the finger domain is very close to the environment of residue 677 (Figure 11C) [45,46]. Interestingly, it was shown in the literature that the L670M change conferred resistance to aphidicolin through an indirect effect on the binding of the template backbone. However, position 670 is located at the extremity of the other α helix forming the finger domain and no resistance to aphidicolin was observed with the A677T. Therefore, the effects of A677T and L670M on the sensitivity to DNA pol inhibitors are not similar.

The residues L510 and L511 are also present in the N-terminal domain close to the residues K503 and L506 that face position 677 (Figure 11D). The amino acid changes L510S and L511F were identified in the drug-resistant selected clones (Figure 11E,F). These two changes resulted in a dramatic modification of the residue’s physicochemical properties. For instance, Leu-to-Ser change at position 510 induced a decrease in the side chain volume from 166.7 Å3 to 89 Å3, while Leu-to-Phe change at position 511 added a very large residue close to the hydrophobic L507 and W112.

**Figure 12:**
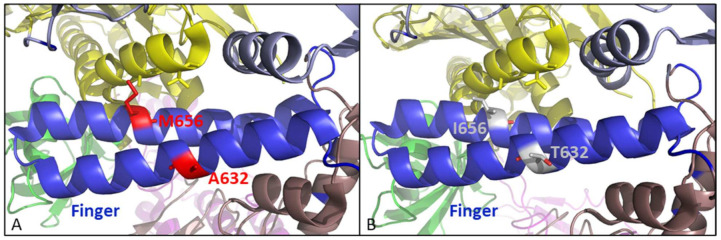
(**A**) Finger domain, represented in blue, is the location of two amino acid changes identified in position A632 and M656 (represented in red). (**B**) The amino acid changes, represented in grey, might involve differences in positioning of the finger domain for proper recognition of the incoming substrate. The images were generated using PyMol Delano Software, version 0.99rc6.

The A632 and M656 are in the middle of the two α helices that compose the finger domain (Figure 12A). It is highly probable that changes in these two positions may affect the polymerase function of poxvirus DNApol, and the identified amino acid changes A632T and M656I might be involved in the drug-resistant phenotype (Figure 12B). Amino acid changes R577G, L696S, and A705T are found in the palm domain of clones selected for resistance to HPMPC (R577G) and HPMPDAP (L696S and A705T), suggesting that the polymerase activity is affected by these variations (Figure 12A). This could be associated with an indirect effect on the binding of the template backbone in the elongation site, as proposed for other amino acid changes located in the palm domain such as A684V/T or T688A.

## 4. Discussion

In this report, we describe the isolation and characterization of DNA pol mutations arising following pressure with different DNA pol inhibitors, encompassing several classes of ANPs, the inhibitor of pyrophosphate exchange PAA, and the fungal tetracyclic diterpenoid aphidicolin (a competitive inhibitor of dCTP and non-competitive inhibitor regarding dATP, dGTP, and dTTP. A schema with the mutations linked to altered drug susceptibility previously described and those found in the present study is depicted in Figure S4. A summary of the phenotypic characteristics of these DNA polymerase mutants is given in Table S4.

Investigations into the anti-poxvirus activity of ANPs have demonstrated that these compounds inhibit viral DNA chain extension [32,47]. In addition, the incorporation of cidofovir (HPMPC) into DNA completely inhibits the associated 3′-to-5′ exonuclease activity. Because of the inhibition of the proofreading exonuclease activity, cidofovir misincorporation could also lead to replication errors during poxvirus DNA synthesis [47]. The proofreading exonuclease activity of poxvirus DNA pols plays a crucial role in minimalizing replication errors and was demonstrated to play a key role in promoting viral genetic recombination during infection, occurring at extraordinarily high levels in poxviruses [48].

Therefore, it is not surprising that we have identified several mutations in the 3′-5′ exonuclease arising relatively fast in the process of drug-resistance selection using different ANPs. This suggests that inhibition of the 3′-5′ exonuclease activity is a common feature of various ANPs. As shown in Figure S4, most of the mutant viruses arising under pressure of cidofovir and other HPMPs and of HPMPO-DAPy acquire the A314/V or the novel change M313I, which could be linked to altered drug susceptibility. Ala-314 and Met-313 can be considered as hot spots for ANP’s resistance as changes at one of these positions were found in various independent selection procedures using different ANPs, including HPMPC, cHPMPC, HPMPA, HPMPDAP, or HPMPO-DAPy. Ala-314 and Met-313 map to the prominent β-finger domain (residues 299–319) located in the exonuclease of the poxvirus DNA pol, a structural element important for switching between elongation and editing modes [45]. The β-finger establishes contact with the template strand during elongation and intervenes in strand separation needed for proofreading [45]. Thus, the exonuclease domain in E9L harbors the main ANP-resistant mutation A314T/V that enhances the excision capacity of nucleotide analogues, such as ANPs, from the 3′ ends of duplex DNA [48]. It is expected that the M313I change may have a similar effect as the A314T/V mutation.

The early and repeated recovery of the A314T/V or M313I in the 3′-5′ exonuclease domain in several independent selections with HPMP and HPMPO derivatives, suggests that these substitutions are probably the primary determinant of resistance. These changes are predicted to enhance drug excision from DNA. Substitutions located in the polymerase domain promoted drug resistance and generally appeared in the backbone of a virus with a 3′-5′ exonuclease mutation. Mutations in the polymerase domain might enhance the incorporation of the ANPs active forms during nucleotide selection. Acquisition of the A314T/V or M313I changes was accompanied by a hypersensitivity to PAA and PMEO-DAPy, suggesting a reduced efficiency of DNA replication. The emergence of mutations in the DNA polymerase domain could be regarded as compensatory mutations that result in higher levels of resistance to HPMP compounds and no alterations in the sensitivity to PAA and PMEO-DAPy.

HPMPA and HPMPC have structural similarities and were shown to inhibit the 3′-5′ proofreading activity of VACV DNA polymerase, explaining why HPMPC-R and HPMPA-R viruses exhibit cross-resistance and acquire mutations in the 3′-to-5′ exonuclease domain. However, HPMPApp, in contrast to HPMPCpp, is a good substrate for the viral enzyme with Km and Vmax parameters equivalent to those of dATP and does not act as a functional chain terminator. Furthermore, HPMPA can be converted to (S)-HPMPApp to a greater degree than HPMPC, which is metabolized to HPMPCpp. Although both drugs can be incorporated into DNA, HPMPA might have a higher likelihood to be incorporated into an irreversible DNA lesion [32].

The 3-deaza-HPMPA derivatives did not follow the same drug-susceptibility profile as the other HPMP’s. Notably, mutations selected under PMEO-DAPy (G372C in the 3′-5′ exonuclease domain and E856 in the polymerase domain) conferred cross-resistance to 3-deaza-HPMPA compounds but not to other HPMP’s. For these substitutions, no cross-resistance or cross-hypersensitivity with PAA was noted. These findings highlight important differences in the mechanism of inhibition of poxvirus DNA polymerases by different ANPs.

Three changes (C356Y, G372D, and G380S) in the VACV DNA polymerase have been previously identified following selective pressure with PAA [44]. As well as conferring resistance to PAA, the C356Y, G372D, and G380S mutations led to a slight cross-resistance to AraC, and hypersensitivity to aphidicolin. Addition of the F171S substitution to any of these three mutations augmented levels of resistance to AraC resistance and the hypersensitivity to aphidicolin but produced no further alteration in the mutation frequency. The C356Y, G372D, and G380S PAA-R mutations map to the poxvirus-specific insert 2 domain of the DNA polymerase, having an indirect effect on the interaction of insert 2 with the finger domain, which might influence the binding of PAA in the deoxynucleotide triphosphate (dNTP) binding site. Since PAA is a competitive inhibitor of pyrophosphate exchange), PAA binds to the PPi-binding region of the dNTP-binding site, and the tight cluster of residues 356, 372, and 380 are expected to have a close relationship with pyrophosphate exchange [34]. Only one out of the four mutants (i.e., F354L) we selected under PAA map to the poxvirus-specific insert 2 domain and, hence, F354L is expected to have a similar effect on the poxvirus DNA polymerase as proposed for the mutations C356Y, G372D, and G380S [36,45]. It is worth mentioning that G372D has been formerly linked to PAA-R [44], while we found that G372C was not linked to PAA-R but to resistance to PMEO-DAPy, AraC, and 3-deaza-HPMPA. This points to the utility of determining the phenotype for all novel mutations as different amino acid substitutions at a specific position do not always have the same consequences on a drug-susceptibility profile.

Our data indicate that several novel changes in the poxvirus DNA polymerase are most likely linked to altered drug susceptibility. The discovery of natural mutants or the construction of recombinant viruses bearing each of the new mutations here described alone or in combination will be of importance to confirm our findings. Similarly, future research should focus on the association of these mutations with a (anti)mutator phenotype and with their impact on virus pathogenicity, as previously described mutations emerging under cidofovir or HPMPDAP were related to reduced pathogenicity in vivo and to a mutator phenotype [37,41,42]. Viruses selected for resistance to HPMPC encoding the A684V substitution mutation but not the A314T change exhibited a mutator phenotype [37]. The S851Y substitution, selected under HPMPDAP pressure, was also able to confer a spontaneous mutator phenotype [41]. Thus, drug-resistant mutations mapping to the putative DNA polymerase domain also create a mutator phenotype. In contrast, three substitutions associated with PAA resistance in the 3′-5′ exonuclease domain (C356T, G372D, and G380S) have been related to a decrease in frequency of the emergence of spontaneous mutations (antimutator phenotype) [34,43].

Previous studies have concluded that mutations conferring PAA resistance (C356T, G372D, and G380S) are linked to aphidicolin hypersensitivity. Vice versa, the A498V and A498T aphidicolin-R mutations showed, respectively, moderate and high sensitivity to PAA and moderate hypersensitivity to AraC [43], but the mechanism behind this mutual effect is not understood [34]. PAA-R and aphidicolin’s mutual effect was also found for the mutants selected under PAA in this study, F354L (PAA-R, AraC-R, PMEO-DAPy-R, and aphidicolin-hs), A684V, and 117ins GSPD + E792D changes (PAA-R, PMEO-DAPy-R, and aphidicolin-hs). In contrast, the Y332H mutation conferred only resistance to PAA. Notably, the A314V mutation, but not the A314T, alone or in combination with other DNA polymerase mutations (even in combination with the PPA-R mutation A684V), was associated with hypersensitivity to PAA, aphidicolin, and AraC as well as to PMEO-DAPy (Table S4), pointing to a dominant effect of the A314V substitution, which was not the case for the A314T change.

Our studies also highlight differences between the HPMP and HPMPO-DAPy derivatives on one side and PMEO-DAPy on the other side, meaning that these compounds have a different mode of interaction with the DNA polymerase. These findings agree with previous reports in herpesviruses highlighting the difference between HPMP and PME compounds [49,50,51].

In a previous study, we have investigated not only the viral DNA polymerase gene (*E9L*), but also the two genes *A20R* and *D4R* coding for the heterodimeric processivity factor of the E9L protein [42]. (S)-HPMPDAP resistance in CMLV was related to mutations in the viral DNA polymerase, but no allele changes in the *A20R* and *D4R* genes were identified [42]. However, sequencing of HPMPC-R MPXV revealed the amino acid change S126L in *A20R*, but the involvement of this change in drug resistance was not examined [38]. Further research should focus on the analysis of the DNA polymerase accessory proteins to elucidate whether changes arise following pressure with ANPs.

Brincidofovir, the intracellular prodrug of cidofovir, received the Orphan Drug Designation from the FDA for the treatment of smallpox. Therefore, a deep understanding of the problem of acquired drug resistance is needed as a first step in evaluating potential difficulties in treating infections caused by viruses bearing such mutations that may arise in the clinic. A good knowledge of the molecular genetic properties and drug-susceptibility patterns of DNA polymerase mutants is also necessary if one wants to develop other drugs directed to this important viral target.

In summary, the mutational patterns associated with resistance to each anti-poxvirus agent appeared to be quite diverse, and cross-resistance patterns within the different classes of DNA polymerase inhibitors (as well as within different ANPs) are complicated. Our studies pave the way for future research in poxvirus drug-resistance and highlight the complex pattern of drug resistance, being the effects of combinations of mutations in the DNA polymerase that are difficult to predict. Our findings may also help in choosing alternative therapies for drug-resistant poxviruses bearing specific changes in the DNA polymerase that may eventually emerge in the clinical setting under novel DNA polymerase inhibitors.

## Figures and Tables

**Table 1:** Identification of amino acid changes in the viral DNA polymerase following selective pressure with HPMPA, cHPMPA, and cHPMPC in VACV Lederle strain.

Drug	Amino Acid Changes in Viral DNA Polymerase			
	**Known Natural Occurring Genetic Polymorphisms**	**Known Drug-Resistant Mutations (Dark Blue) and Novel Mutations (Yellow)**		
HPMPA	T845M, G857R, 936–938 ANV	A314T	—	—
cHPMPA	T845M, G857R, 936–938 ANV	—	A353V	S851Y
cHPMPC	T845M, G857R, 936–938 NΔG	A314T	—	—

The complete DNA pol (E9L gene) was genotyped for one of the viral clones isolated from each selection procedure with the indicated drug. The presence of the mutations was verified in additional viral clones, encompassing 8 viral clones selected under HPMPA and cHPMPA and 9 viral clones under cHPMPC.

**Table 2:** Identification of amino acid changes in the CPXV Brighton strain DNA polymerase following selective pressure with HPMPC, HPMPDAP, and HPMPO-DAPy.

Drug	Amino Acid Changes in Viral DNA Polymerase					
	**Known Natural Occurring Genetic Polymorphisms**	**Known Drug-R Mutations (Dark Blue) and Novel Mutations (Yellow)**				
HPMPC (clones 1, 2, 4, 5, 6, 7)	—	A314V	—	A684V	—	—
HPMPDAP (clones 1, 2, 3)	—	—	Ins408 (amino acids KDIICKVIH)	—	—	L924F
HPMPDAP (clones 4, 5, 6, 7, 8)	—	—	—	—	—	L924F
HPMPO-DAPy (clones 1, 4, 5, 6, 7)	—	—	—	—	Ins851L	—

**Table 3:** Identification of amino acid changes in the VACV DNA polymerase following selective pressure with the ANPs HPMPC, HPMPDAP, and HPMPO-DAPy starting from four plaque-purified clones, denoted VV1, VV2, VV8, and VV11, isolated from the parental VACV Lederle strain.

A. VACV Mutants Selected under Pressure of HPMPC (Cidofovir).											
**Virus**	**Clone**	**Amino Acid Changes in Viral DNA Polymerase**									
		**Known Natural Occurring Genetic Polymorphisms**	**Known Drug-Resistant Mutations** (**Dark Blue**) **and Novel Mutations** (**Yellow**)								
VV1	clone 3	R97H, T845M, G857R, 936–938 NΔG	—			A314T	—		—	A684V	
VV2	clone 2	W8C, 936–938 ANV	M313I			—	R577G		A677T	A684V	
VV8	clone 1	R246Q, G857R, 936–938 ANV	—			A314V	L510S		—	A684V	
VV11	clone 1	L90S, R235L, T845M, G857R, S898T, 936–938 NΔG	—			A314T	—		M656I	A684V	
B. VACV Mutants Selected under Pressure of HPMPDAP.											
**Virus**	**Clone**	**Amino Acid Changes in Viral DNA Polymerase**									
		**Known Natural Occurring Genetic Polymorphisms**	**Known Drug-Resistant Mutations** (**Dark Blue**) **and Novel Mutations** (**Yellow**)								
VV1	clone 1	R23G, L420S, T845M, G857R, 936–938 NΔG	A314V			—	T500I	—		T688A	—
VV2	clone 3	T71I, T845M, G857R, 936–938 NΔG	—			D480Y	—	A684V		—	A705T
VV8	clone 1	R246Q, T845M, G857R, 936–938 NΔG	A314T			—	—	A684V		—	—
VV11	clone 1	I68T, L420S, T845M, G857R, C865S, 936–938 NΔG	A314T			—	—	A684V		—	L696S
C. VACV Mutants Selected under Pressure of HPMPO-DAPy.											
**Virus**	**Clone**	**Amino Acid Changes in Viral DNA Polymerase**									
		**Known Natural Occurring Genetic Polymorphisms**	**Known Drug-Resistant Mutations** (**Dark Blue**) **and Novel Mutations** (**Yellow**)								
VV1	clone 3	T845M, G857R, 936–938 NΔG	**—**	**—**	—	—	—	—	A632T	—	S851Y
VV2	clone 3	T845M, G857R, 936–938 NΔG	R155S	M313I	-	R407C	L511F	—	—	A684V	—
VV8	clone 1	R246Q, T845M, G857R, 936–938 NΔG	—	M313I	—	—	—	A613T	—	A684V	—
VV11	clone 1	T845M, G857R, 936–938 NΔG	G138E	—	A314T	—	—	—	—	—	—

**Table 4:** Identification of amino acid changes in the viral DNA polymerase following selective pressure with the pyrophosphate analogue phosphonoacetic acid (PAA) (A) or the acyclic nucleoside phosphonate PMEO-DAPy (B).

A. VACV and CPXV Mutants Selected under Pressure of PAA.							
**Virus**	**Clone**	**Amino Acid Changes in Viral DNA Polymerase**					
		**Known Natural Occurring Genetic Polymorphisms**	**Known Drug-Resistant Mutations** (**Dark Blue**) **and Novel Mutations** (**Yellow**)				
VV1	clone 1	T845M, G857R, 936–938 NΔG	—	—	F354L	—	—
VV2	clone 1	T845M, G857R, 936–938 NΔG	—	—	—	A684V	—
VV8	clone 1	R246Q, G857R	117Ins. GISPD	—	—	—	E792D
VV11	clone 1	L420S, T845M, G857R, 936–938 NΔG	—	Y332H	—	—	—
B. VACV Mutants Selected under Pressure of PMEO-DAPy.							
**Virus**	**Clone**	**Amino Acid Changes in Viral DNA Polymerase**					
		**Known Natural Occurring Genetic Polymorphisms**	**Novel Drug-Resistant Mutations** (**Yellow**)				
VV1	clone 1	L420, T845M, 936–938 ANV	G372C		—		
VV2	clone 1	T845M, 936–938 NΔG	—		E856K		

## Data Availability

All data are available within the article and Supplementary Files, or from the authors upon request.

## Supplementary Materials

The following are available online at https://www.mdpi.com/article/10.3390/biomedicines10030580/s1, Figure S1: Annealing of primers used for the amplification of the E9L gene, Figure S2: Primers used for sequencing of the E9L gene, Figure S3: Genotyping (sequencing of the E9L gene, DNA polymerase) and phenotyping of viral clones derived from VACV VV1 emerging under pressure with HPMPC, Figure S4: Diagram of poxvirus DNA polymerase with previously described mutations known to be associated with altered drug susceptibility and mutations reported in the present study. Table S1: Starting and ending drug concentrations used for the drug escalations, Table S2: Genotyping (sequencing of the E9L gene, DNA polymerase) of viral clones derived from VACV VV2 emerging under pressure with HPMPC, Table S3: Genotyping (sequencing of the E9L gene, DNA polymerase) of viral clones derived from VACV VV11 emerging under pressure with HPMPC, Table S4: Summary of the known and new DNA polymerase mutations in poxviruses associated with altered drug sensitivity and their putative mode of action.

## References

[1] Foster S.A., Parker S., Lanier R. (2017). The Role of Brincidofovir in Preparation for a Potential Smallpox Outbreak. Viruses.

[2] Jacobs B.L., Langland J.O., Kibler K.V., Denzler K.L., White S.D., Holechek S.A., Wong S., Huynh T., Baskin C.R. (2009). Vaccinia virus vaccines: Past, present and future. Antivir. Res..

[3] Fenner F. (1993). Smallpox: Emergence, global spread, and eradication. Hist. Philos. Life Sci..

[4] Breman J.G., Arita I. (1980). The Confirmation and Maintenance of Smallpox Eradication. N. Engl. J. Med..

[5] Melamed S., Israely T., Paran N. (2018). Challenges and Achievements in Prevention and Treatment of Smallpox. Vaccines.

[6] Grosenbach D.W., Honeychurch K., Rose E.A., Chinsangaram J., Frimm A., Maiti B., Lovejoy C., Meara I., Long P., Hruby D.E. (2018). Oral Tecovirimat for the Treatment of Smallpox. N. Engl. J. Med..

[7] Yang G., Pevear D.C., Davies M.H., Collett M.S., Bailey T., Rippen S., Barone L., Burns C., Rhodes G., Tohan S. (2005). An Orally Bioavailable Antipoxvirus Compound (ST-246) Inhibits Extracellular Virus Formation and Protects Mice from Lethal Orthopoxvirus Challenge. J. Virol..

[8] Duraffour S., Lorenzo M.M., Zöller G., Topalis D., Grosenbach D., Hruby D.E., Andrei G., Blasco R., Meyer H., Snoeck R. (2015). ST-246 is a key antiviral to inhibit the viral F13L phospholipase, one of the essential proteins for orthopoxvirus wrapping. J. Antimicrob. Chemother..

[9] Chan-Tack K., Harrington P., Bensman T., Choi S.-Y., Donaldson E., O’Rear J., McMillan D., Myers L., Seaton M., Ghantous H. (2021). Benefit-risk assessment for brincidofovir for the treatment of smallpox: U.S. Food and Drug Administration’s Evaluation. Antivir. Res..

[10] Alvarez-Cardona J.J., Whited L.K., Chemaly R.F. (2020). Brincidofovir: Understanding its unique profile and potential role against ade-novirus and other viral infections. Future Microbiol..

[11] Delaune D., Iseni F. (2020). Drug Development against Smallpox: Present and Future. Antimicrob. Agents Chemother..

[12] Kabuga A.I., El Zowalaty M.E. (2019). A review of the monkeypox virus and a recent outbreak of skin rash disease in Nigeria. J. Med. Virol..

[13] Guagliardo S.A.J., Monroe B., Moundjoa C., Athanase A., Okpu G., Burgado J., Townsend M.B., Satheshkumar P.S., Epperson S., Doty J.B. (2020). Asymptomatic Orthopoxvirus Circulation in Humans in the Wake of a Monkeypox Outbreak among Chimpanzees in Cameroon. Am. J. Trop. Med. Hyg..

[14] Reynolds M.G., Doty J.B., Mccollum A.M., Olson V.A., Nakazawa Y. (2018). Monkeypox re-emergence in Africa: A call to expand the concept and practice of One Health. Expert Rev. Anti-Infect. Ther..

[15] Di Giulio D.B., Eckburg P.B. (2004). Human monkeypox: An emerging zoonosis. Lancet Infect. Dis..

[16] Shisler J.L. (2015). Immune Evasion Strategies of Molluscum Contagiosum Virus. Adv. Virus Res..

[17] Romero R.M., Navarrete-Dechent C., Downey C. (2019). Molluscum contagiosum: An update and review of new perspectives in etiology, diagnosis, and treatment. Clin. Cosmet. Investig. Dermatol..

[18] Abrahao J.S., Campos R.K., Trindade Gde S., Guimaraes da Fonseca F., Ferreira P.C., Kroon E.G. (2015). Outbreak of severe zoonotic vaccinia virus infection, Southeastern Brazil. Emerg. Infect. Dis..

[19] Lu B., Cui L.-B., Gu M.-H., Shi C., Sun C.-W., Zhao K.-C., Bi J., Tan Z.-M., Guo X.-L., Huo X. (2019). Outbreak of Vaccinia Virus Infection from Occupational Exposure, China, 2017. Emerg. Infect. Dis..

[20] Lima M.T., Oliveira G., Afonso J.A.B., Souto R.J.C., De Mendonça C.L., Dantas A.F.M., Abrahao J.S., Kroon E.G. (2019). An Update on the Known Host Range of the Brazilian Vaccinia Virus: An Outbreak in Buffalo Calves. Front. Microbiol..

[21] Silva N.I.O., De Oliveira J.S., Kroon E.G., Trindade G.D.S., Drumond B.P. (2020). Here, There, and Everywhere: The Wide Host Range and Geographic Distribution of Zoonotic Orthopoxviruses. Viruses.

[22] Wollenberg A., Vogel S., Sã¡rdy M., Glos K., Korting H., Ruzicka T. (2012). The Munich Outbreak of Cutaneous Cowpox Infection: Transmission by Infected Pet Rats. Acta Derm. Venereol..

[23] Snoeck R., Holý A., Dewolf-Peeters C., Oord J.V.D., De Clercq E., Andrei G. (2002). Antivaccinia Activities of Acyclic Nucleoside Phosphonate Derivatives in Epithelial Cells and Organotypic Cultures. Antimicrob. Agents Chemother..

[24] Keith K.A., Wan W.B., Ciesla S.L., Beadle J.R., Hostetler K.Y., Kern E.R. (2004). Inhibitory Activity of Alkoxyalkyl and Alkyl Esters of Cidofovir and Cyclic Cidofovir against Orthopoxvirus Replication In Vitro. Antimicrob. Agents Chemother..

[25] Hocková D., Holý A., Masojídková M., Andrei G., Snoeck R., De Clercq E., Balzarini J. (2003). 5-Substituted-2,4-diamino-6-[2-(phosphonomethoxy)ethoxy]pyrimidines-acycli c nucleoside phosphonate analogues with an-tiviral activity. J. Med. Chem..

[26] De Clercq E., Holy A. (2005). Acyclic nucleoside phosphonates: A key class of antiviral drugs. Nat. Rev. Drug Discov..

[27] Duraffour S., Snoeck R., Krecmerová M., Oord J.V.D., De Vos R., Holý A., Crance J.-M., Garin D., De Clercq E., Andrei G. (2007). Activities of Several Classes of Acyclic Nucleoside Phosphonates against Camelpox Virus Replication in Different Cell Culture Models. Antimicrob. Agents Chemother..

[28] Dal Pozzo F., Andrei G., Holy A., Van Den Oord J., Scagliarini A., De Clercq E., Snoeck R. (2005). Activities of acyclic nucleoside phosphonates against Orf virus in human and ovine cell monolayers and organotypic ovine raft cultures. Antimicrob Agents Chemother..

[29] Lebeau I., Andrei G., Dal Pozzo F., Beadle J.R., Hostetler K.Y., De Clercq E., Van Den Oord J., Snoeck R. (2006). Activities of alkoxyalkyl esters of cidofovir (CDV), cyclic CDV, and (S)-9-(3-hydroxy-2-phosphonylmethoxypropyl)adenine against orthopoxviruses in cell monolayers and in organotypic cultures. Antimicrob Agents Chemother..

[30] Dal Pozzo F., Andrei G., Lebeau I., Beadle J.R., Hostetler K.Y., De Clercq E., Snoeck R. (2007). In vitro evaluation of the anti-orf virus activity of alkoxyalkyl esters of CDV, cCDV and (S)-HPMPA. Antiviral Res..

[31] Hostetler K.Y. (2009). Alkoxyalkyl prodrugs of acyclic nucleoside phosphonates enhance oral antiviral activity and reduce toxicity: Current state of the art. Antivir. Res..

[32] Magee W.C., Aldern K.A., Hostetler K.Y., Evans D.H. (2008). Cidofovir and (S)-9-[3-Hydroxy-(2-Phosphonomethoxy)Propyl]Adenine Are Highly Effective Inhibitors of Vaccinia Virus DNA Polymerase When Incorporated into the Template Strand. Antimicrob. Agents Chemother..

[33] Zahn K., Tchesnokov E.P., Götte M., Doublié S. (2011). Phosphonoformic Acid Inhibits Viral Replication by Trapping the Closed Form of the DNA Polymerase. J. Biol. Chem..

[34] Czarnecki M.W., Traktman P. (2017). The vaccinia virus DNA polymerase and its processivity factor. Virus Res..

[35] Andrei G., Snoeck R. (2010). Cidofovir Activity against Poxvirus Infections. Viruses.

[36] Sèle C., Gabel F., Gutsche I., Ivanov I., Burmeister W.P., Iseni F., Tarbouriech N. (2012). Low-Resolution Structure of Vaccinia Virus DNA Replication Machinery. J. Virol..

[37] Andrei G., Gammon D.B., Fiten P., De Clercq E., Opdenakker G., Snoeck R., Evans D.H. (2006). Cidofovir resistance in vaccinia virus is linked to diminished virulence in mice. J. Virol..

[38] Farlow J., Ichou M.A., Huggins J., Ibrahim S. (2010). Comparative whole genome sequence analysis of wild-type and cidofovir-resistant monkeypoxvirus. Virol. J..

[39] Becker M.N., Obraztsova M., Kern E.R., Quenelle D.C., Keith K., Prichard M.N., Luo M., Moyer R.W. (2008). Isolation and characterization of cidofovir resistant vaccinia viruses. Virol. J..

[40] Kornbluth R.S., Smee D.F., Sidwell R.W., Snarsky V., Evans D.H., Hostetler K.Y. (2006). Mutations in the E9L polymerase gene of cidofo-vir-resistant vaccinia virus strain WR are associated with the drug resistance phenotype. Antimicrob. Agents Chemother..

[41] Gammon D.B., Snoeck R., Fiten P., Krecmerová M., Holyý A., De Clercq E., Opdenakker G., Evans D., Andrei G. (2008). Mechanism of Antiviral Drug Resistance of Vaccinia Virus: Identification of Residues in the Viral DNA Polymerase Conferring Differential Resistance to Antipoxvirus Drugs. J. Virol..

[42] Duraffour S., Andrei G., Topalis D., Krečmerová M., Crance J.-M., Garin D., Snoeck R. (2012). Mutations Conferring Resistance to Viral DNA Polymerase Inhibitors in Camelpox Virus Give Different Drug-Susceptibility Profiles in Vaccinia Virus. J. Virol..

[43] Taddie J.A., Traktman P. (1991). Genetic characterization of the vaccinia virus DNA polymerase: Identification of point mutations conferring altered drug sensitivities and reduced fidelity. J. Virol..

[44] Taddie J.A., Traktman P. (1993). Genetic characterization of the vaccinia virus DNA polymerase: Cytosine arabinoside resistance re-quires a variable lesion conferring phosphonoacetate resistance in conjunction with an invariant mutation localized to the 3′-5′ exonuclease domain. J. Virol..

[45] Tarbouriech N., Ducournau C., Hutin S., Mas P.J., Man P., Forest E., Hart D.J., Peyrefitte C.N., Burmeister W.P., Iseni F. (2017). The vaccinia virus DNA polymerase structure provides insights into the mode of processivity factor binding. Nat. Commun..

[46] DeFilippes F.M. (1989). Site of the base change in the vaccinia virus DNA polymerase gene which confers aphidicolin resistance. J. Virol..

[47] Magee W.C., Hostetler K., Evans D.H. (2005). Mechanism of Inhibition of Vaccinia Virus DNA Polymerase by Cidofovir Diphosphate. Antimicrob. Agents Chemother..

[48] Gammon D.B., Evans D.H. (2009). The 3′-to-5′ Exonuclease Activity of Vaccinia Virus DNA Polymerase Is Essential and Plays a Role in Promoting Virus Genetic Recombination. J. Virol..

[49] Balzarini J., Pannecouque C., Naesens L., Andrei G., Snoeck R., De Clercq E., Hockova D., Holy A. (2004). 6-[2-(Phosphonomethoxy)alkoxy]-2,4-diaminopyrimidines: A New Class of Acyclic Pyrimidine Nucleoside Phosphonates with Antiviral Activity. Nucleotides Nucleic Acids.

[50] Andrei G., Fiten P., Froeyen M., De Clercq E., Opdenakker G., Snoeck R. (2007). DNA polymerase mutations in drug-resistant herpes simplex virus mutants determine in vivo neurovirulence and drug-enzyme interactions. Antivir. Ther..

[51] Andrei G., Topalis D., Fiten P., McGuigan C., Balzarini J., Opdenakker G., Snoeck R. (2011). In Vitro-Selected Drug-Resistant Varicella-Zoster Virus Mutants in the Thymidine Kinase and DNA Polymerase Genes Yield Novel Phenotype-Genotype Associations and Highlight Differences between Antiherpesvirus Drugs. J. Virol..

