# Supplementary material for: Poxviruses Bearing DNA Polymerase Mutations Show Complex Patterns of Cross-Resistance
Source: Biomedicines. 2022 Mar 1;10(3):580. doi: 10.3390/biomedicines10030580 (PMC8945813; doi:10.3390/biomedicines10030580)
Supplement: Supplementary file 1 [file biomedicines-10-00580-s001.zip › biomedicines-1513598-supplementary.pdf]

biomedicines

MDPI

Figure S1. Annealing of primers used for the amplification of the E9L gene. The numbers indicate the nucleotide position in the VACV genome. In brown is highlighted the nucleotide sequencing encoding the DNA polymerase. Forward (in green) and reverse (in yellow) primers used for PCR amplification are as well highlighted.

```
53581 tctaatggat aaactgaatc taacaaagag cgacgtacaa ctgttgtaaa ttatttatg
53641 cttcgtaaaa tgtaggtttt gaaccaaaca ttctttcaaa gaatgagatg cataaaactt
53701 tattatccaa tagattgact atttcggacg tcaatcgttt aaagtaaact tcgtaaaata
53761 ttctttgatc actgccgagt ttaaaacttc tatcgataat tgtttcatat gttttaatat
53821 ttacaagttt tttggtccat ggtacattag ccggacaaat atatgcaaaa taatatcgtt
53881 ctccaagttc tatagtttct ggattatttt tattatattc agtaaccaaa tacatattag
53941 ggttatctgc ggatttataa tttgagtgat gcattcgact caacataaat aattctagag
54001 gagacgatct actatcaaat tcggatcgta aatctgtttc taaagaacgg agaatatcta
54061 tacatacctg attagaattc atccgtcctt cagacaacat ctcagacagt ctggtcttgt
54121 atgtcttaat catattctta tgaaacttgg aaacatctct tctagtttca ctagtacctt
54181 tattaattct ctcaggtaca gattttgaat tcgacgatgc cgagtatttc atcgttgtat
54241 atttcttctt cgattgcata atcagattct tatataccgc ctcaaactct attttaaaat
54301 tattaaacaa tactctatta ttaatcagtc gttctaactc ctttgctatt tctatggact
54361 tatctacatc ttgactgtct atctctgtaa acacggagtc ggtatctcca tacacgctac
54421 gaaaacgaaa tctgtaatct ataggcaacg atgttttcac aatcggatta atatctctat
54481 cgtccatata aaatggatta cttaatggat tggcaaaccg taacataccg ttagataact
54541 ctgctccatt tagtaccgat tctagataca agatcattct acgtcctatg gatgtgcaac
54601 tcttagccga agcgtatgag tatagagcac tatttctaaa tcccatcaga ccatatactg
54661 agttggctac tatcttgtac gtatattgca tggaatcata gatggccttt tcagttgaac
54721 tggtagcctg ttttaacatc tttttatatc tggctctctc tgccaaaaat gttcttaata
54781 gtctaggaat ggttccttct atcgatctat cgaaaattgc tatttcagag atgaggttcg
54841 gtagtctagg ttcacaatga accgtaatat atctaggagg tggatatttc tgaagcaaga
54901 gctgattatt tatttcttct tccaatctat tggtactaac aacgacaccg actaatgttt
54961 ccggagatag atttccaaag atacacacat taggatacag actgttataa tcaaagatta
55021 atacattatt actaaacatt ttttgttttg gagcaaatac cttaccgcct tcataaggaa
55081 acttttgttt tgtttctgat ctaactaaga tagttttagt ttccaacaat agctttaaca
55141 gtggaccctt gatgactgta ctcgctctat attcgaatac catggattga ggaagcacat
55201 atgttgacgc acccgcgtct gtttttgttt ctactccata atactcccac aaatactgac
55261 acaaacaagc atcatgaata cagtatctag ccatatctaa agctatgttt agattataat
55321 ccttatacat ctgagctaaa tcaacgtcat cctttccgaa agataattta tatgtatcat
55381 taggtaaagt aggacataat agtacgactt taaatccatt ttcccaaata tctttacgaa
55441 ttactttaca tataatatcc tcatcaacag tcacataatt acctgtggtt aaaacctttg
55501 caaatgcagc ggctttgcct ttcgcgtccg tagtatcgtc accgatgaac gtcatttctc
55561 taactcctct atttaatact ttacccatgc aactgaacgc gttcttggat atagaatcca
55621 atttgtacga atccaatttt tcagattttt gaatgaatga atatagatcg aaaaatatag
55681 ttccattatt gttattaacg tgaaacgtag tattggccat gccgcctact cccttatgac
55741 tagactgatt tctctcataa atacagagat gtacagcttc ctttttgtcc ggagatctaa
55801 agataatctt ctctcctgtt aataactcta gacgattagt aatatatctc agatcaaagt
55861 tatgtccgtt aaaggtaacg acatagtcga acgttagttc caacaattgt ttagctattc
55921 gtaacaaaac tatttcagaa cataaaacta gttctcgttc gtaatccatt tccattagtg
55981 actgtatcct caaacatcct ctatcgacgg cttcttgtat ttcctgttcc gttaacatct
56041 cttcattaat gagcgtaaac aataatcgtt taccacttaa atcgatataa cagtaacttg
56101 tatgcgagat tgggttaata aatacagaag gaaacttctt atcgaagtga cactctatat
56161 ctagaaataa gtacgatctt gggatatcga atctaggtat ttttttagcg aaacagttac
56221 gtggatcgtc acaatgataa catccattgt taatctttgt caaatattgc tcgtccaacg
56281 agtaacatcc gtctggagat atcccgttag aaatataaaa ccaactaata ttgagaaatt
56341 catccatggt ggcattttgt atgctgcgtt tctttggctc ttctatcaac cacatatctg
56401 cgacggagca ttttctatct ttaatatcta gattataact tattgtctcg tcaatgtcta
56461 tagttctcat ctttcccaac ggcctcgcat taaatggagg aggagacaat gactgatata
56521 tttcgtccgt cactacgtaa taaaagtaat gaggaaatcg tataaatacg gtctcaccat
56581 ttcgacatct ggatttcaga tataaaaatc tgttttcacc gtgactttca aaccaattaa
56641 tgcaccgaac atccatttat agaatttaga aatatatttt catttaaatg aatcccaaac
56701 attgggaag agccgtatgg accattattt ttatagtact ttcgcaagcg ggtttagacg
```

The sets of primers used for amplification were:

Primer set 1: 5′-ATAATGGTCCATACGGCTCTTCCC-3′ & 5′-TGGAGCAAATACCTTACCGCCTTC-3′

Primer set 2: 5-AGTCATCAAGGGTCCACTGTTAAAGC-3′ & 5-GATAAACTGAATCTAACAAAGAGCGACG-3′

Figure S2. Primers used for sequencing of the E9L gene.

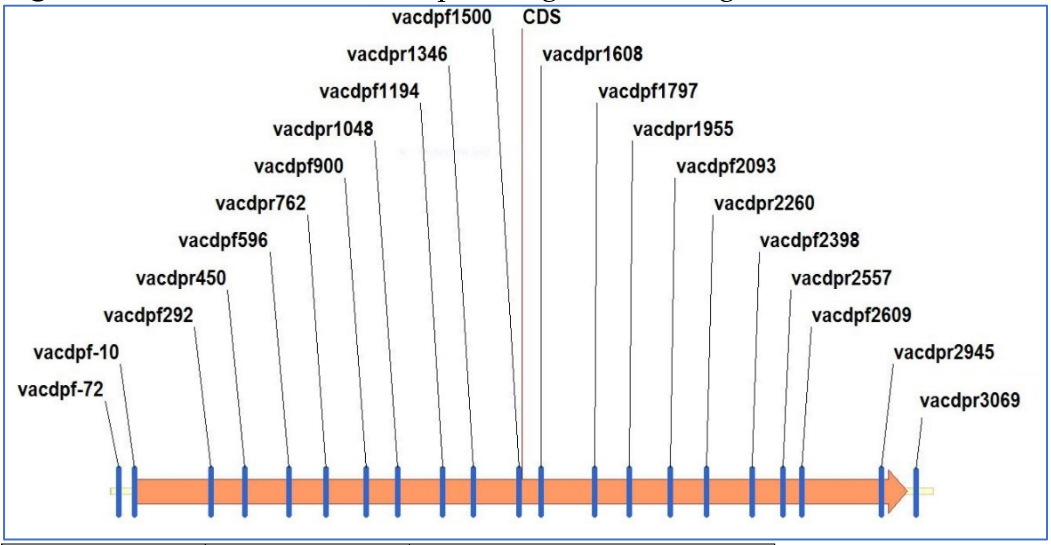

Amplicon	Name	Sequence (5' - 3')
VACDP_F	vacdpf-72	ataatggtccatacggctcttccc
	vacdpF-10	aattctataaatggatgttcggtgc
	vacdpf292	agcatacaaaatgccaccatggatg
	vacdpf596	tgtttacgctcattaatgaagagatg
	vacdpf900	tgagagaaatcagtctagtcataagg
	vacdpf1194	tgaggatattatatgtaaagtaattcg
	vacdpr450	agcgaaacagttacgtggatcgtcac
	vacdpr762	tagttccaacaattgtttagctattcg
	vacdpr1048	atatagaatccaatttgtacgaatcc
	vacdpr1346	agattataatccttatacatctgagc
	vacdpr1608	tggagcaaataccttaccgccttc
VACDP_R	vacdpf1500	agtcatcaagggtccactgttaaagc
	vacdpf1797	tcattgtgaacctagactaccgaacc
	vacdpf2093	tagaatcggtactaaatggagcag
	vacdpf2398	atgcaatcgaagaagaaatatacaacg
	vacdpf2609	gttctttagaaacagatttacgatcc
	vacdpr1955	atggcctttcagttgaactggtag
	vacdpr2260	agtcggtatctccatacacgctacg
	vacdpr2557	tctcagacagtctggtcttgtatgtc
	vacdpR2945	agattgactatttcggacgtcaatcg
	vacdpr3069	gataaactgaatctaacaaagagcgacg

Figure S3. (A) Genotyping (sequencing of the E9L gene, DNA polymerase) and phenotyping of viral clones derived from VACV VV1 emerging under pressure with HPMPC. **(B)** *Drug-susceptibility profile of selected VV1 HPMPC-R viral clones to evaluate the impact of the R97H, A314T, and A684V mutations. Drug-resistance properties of the different viral clones were determined using a CPE reduction assay with HEL fibroblasts. The effects of different drugs on viruses encoding the indicated mutations were determined by calculating the EC_{50} values for the parental wild-type strain and clones bearing the specific mutations. Al least two independent experiments were performed for each test compound. Horizontal lines for each drug and mutant viral clones indicate the mean values ± standard deviation. The fold-resistance (ratio of the EC_{50} for the mutant viruses to the EC_{50} for the corresponding wild-type virus is marked at the top of the graph. VACV viral clones showing a ≥2-fold increase (red) were considered drug-resistant (R) and those with a ≤ 0.5-fold decrease (orange) were considered drug-hypersensitive (hs).*

Virus clones analyzed	DNA pol mutations		
VV1 / HPMPC^{r} clone 1	R97H	A314T	A684V
VV1 / HPMPC^{r} clone 2	R97H	A314T	A684V
**VV1 / HPMPC^{r} clone 3***	R97H	A314T	A684V
VV1 / HPMPC^{r} clone 4	R97H	A314T	A684V
VV1 / HPMPC^{r} clone 5	R97H	A314T	A684V

A

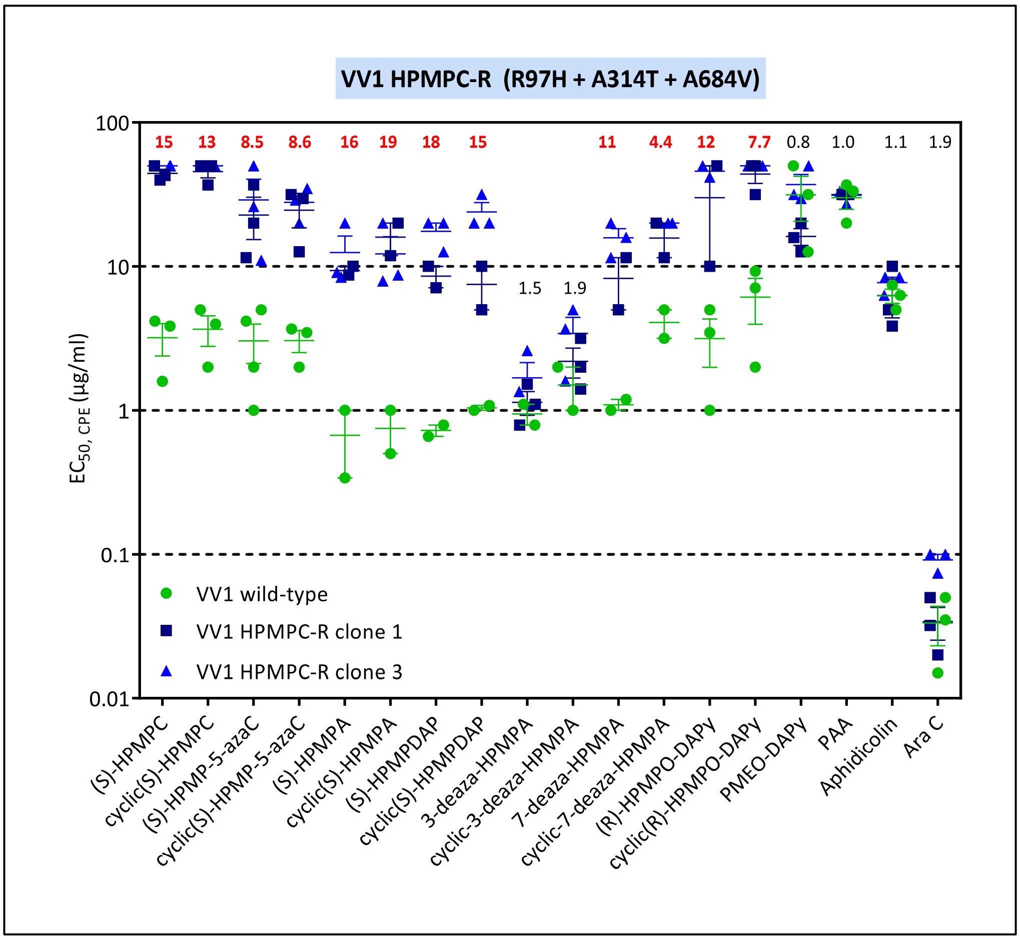

B

Figure S4. Diagram of poxvirus DNA polymerase with previously described mutations known to be associated with altered drug-

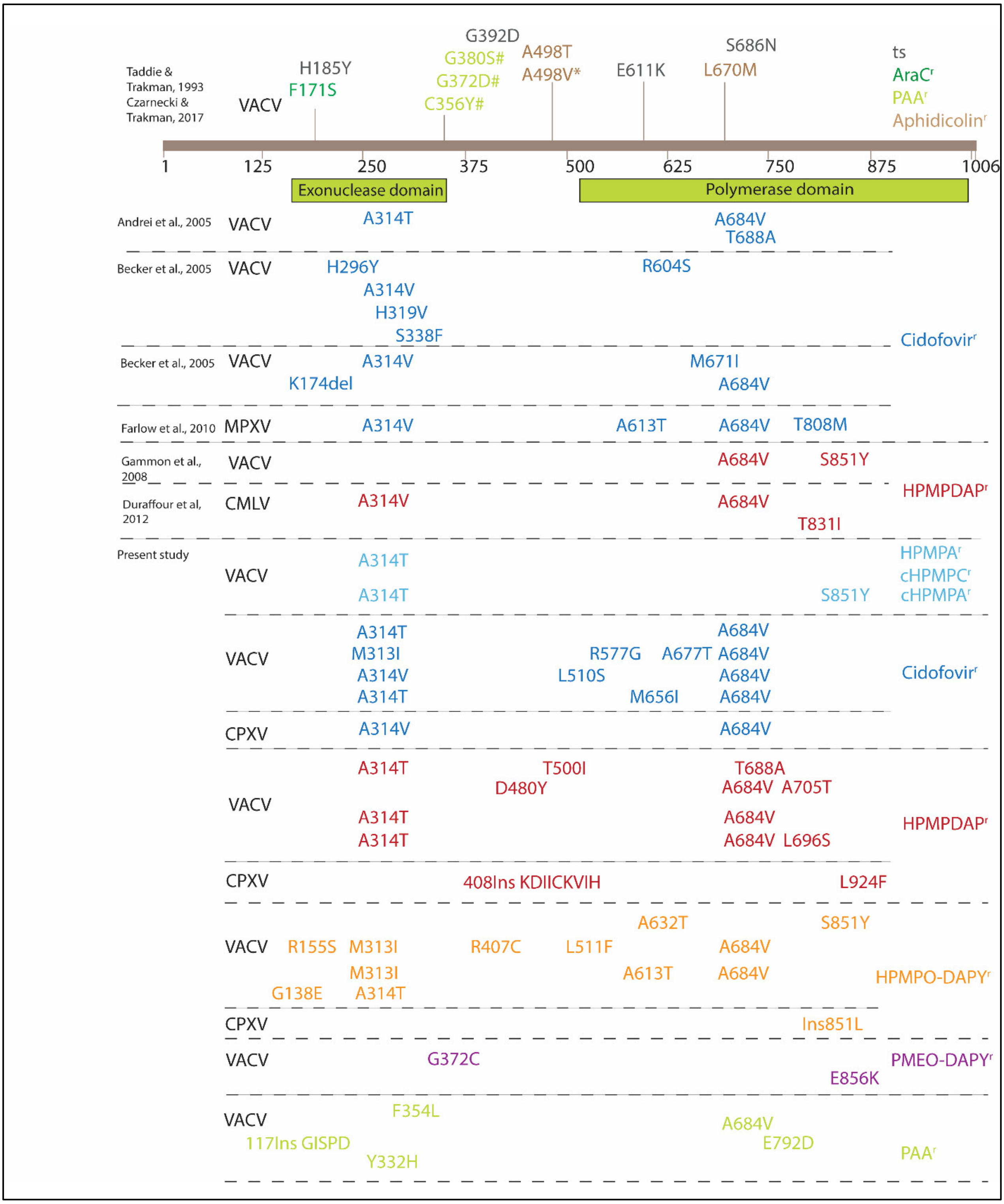

susceptibility and mutations reported in the present study.

Table S1. **Starting and ending drug concentrations used for the drug escalations**

**Drug**	**Starting concentration (µg/ml)**	**End concentration (µg/ml)**
Cidofovir (HPMPC)	1	50
cHPMPC	1	50
HPMPA	0.2	30
cHPMPA	0.2	4
HPMPDAP	1	50
HPMPO-DAPy	1	50
PMEO-DAPy	20	200
PAA	20	150

Table S2. Genotyping (sequencing of the E9L gene, DNA polymerase) of viral clones derived from VACV VV2 emerging under pressure with HPMPC.

**Virus clones analyzed**	**DNA pol mutations**				
VV2 / HPMPC^r clone 1	W8C	M313I	R577G	A677	A684V
**VV2 / HPMPC^r clone 2***	W8C	M313I	R577G	A677T	A684V
VV2 / HPMPC^r clone 3	W8C	M313I	R577G	A677	A684V
VV2 / HPMPC^r clone 4	W8C	M313I	R577G	A677T	A684V
VV2 / HPMPC^r clone 5	W8C	M313I	R577G	A677	A684V

Table S3. Genotyping (sequencing of the E9L gene, DNA polymerase) of viral clones derived from VACV VV11 emerging under pressure with HPMPC.

**Virus clones analyzed**	**DNA pol mutations**					
**VV11 / HPMPC^r clone 1***	L90S	R234L	A314T	M656I	A684V	**S898T**
VV11 / HPMPC^r clone 2	L90S	R234	A314T	M656I	A684V	**S898T**
VV11 / HPMPC^r clone 3	L90	R234	A314T	M656I	A684V	**S898**
VV11 / HPMPC^r clone 4	L90	R234	A314T	M656I	A684V	**S898**
VV11 / HPMPC^r clone 5	L90	R234	A314T	M656I	A684V	**S898**
VV11 / HPMPCr clone 6	L90S	R234	A314T	M656I	A684V	**S898T**
VV11 / HPMPCr clone 7	L90	R234	A314T	M656I	A684V	S898T

Table S4. Summary of the known and new DNA polymerase mutations in poxviruses associated with altered drug-sensitivity and their putative mode of action

**E9L mutation**	**Virus**	**Selected under pressure of:**	**Sensitivity / Hypersensitivity (hs) / resistance**			**Putative mechanism**
			**HPMP's & HPMPO-DAPy's**	**3-deaza-HPMPA HPMP-5-azaC**	**PMEO-DAPy, PAA, aphidicolin & AraC**	
**F171S (44)***	VACV	AraC	Not available	Not available	AraC-R<br>AraA-R<br>Aphidicolin-hs	**F171S** (3′-5′ exonuclease domain, Exo-I): modifying the 3′ nucleotide binding at the exonuclease site (45)
**K174 del (39)**	VACV	Cidofovir (HPMPC)	HPMPC-R - no data availale for other ANPs	Not available	Not available	**K174 del** (3′-5′ exonuclease domain): modifying the 3′ nucleotide binding at the exonuclease site (45)
**H185T (45, 52)**	VACV	*ts*	Not available	Not available	Not available	**H185T** (3′-5′ exonuclease domain): Destabilisation of the 3′-5′ exonuclease domain (45)
**M313I**	VACV	HPMPC<br>HPMPO-DAPy	Low resistance	HPMP-5-azaC-S<br>3-deaza-HPMPA-S	PMEO-DAPy-hs<br>PAA-hs<br>Aphidicolin-S<br>Arac-S	**M313I:** (3′-5′ exonuclease domain, beta-hairpin): Modulation of guidance from elongation to editing mode
**M313I + A613T**	VACV	HPMPO-DAPy	Higher resistance than M313I	HPMP-5-azaC-R<br>3-deaza-HPMPA-S	PMEO-DAPy-hs<br>PAA-hs<br>Aphidicolin-S<br>Arac-S	**A613T** (polymerase domain): Destabilization of insert 3 or of the elongation site
**M313I + A613T + A684V**	VACV	HPMPO-DAPy	Higher resistance than M313I	HPMP-5-azaC-R<br>3-deaza-HPMPA-R	PMEO-DAPy-R<br>PAA-S<br>Aphidicolin-S<br>Arac-R	**A684V** (polymerase domain): indirect effect on the binding of the template backbone in the elongation site (45)
**M313I + R577G + A684V**	VACV	HPMPC	Higher resistance than M313I	HPMP-5-azaC-R<br>3-deaza-HPMPA-S	PMEO-DAPy-hs<br>PAA-S<br>Aphidicolin-hs<br>Arac-hs	**R577G** (polymerase domain) catalytic impairment of the enzyme
**M313I + R577G + A677T + A684V**	VACV	HPMPC	Higher resistance than M313I + R577G + A684V	HPMP-5-azaC-R<br>3-deaza-HPMPA-S	PMEO-DAPy-hs<br>PAA-S<br>Aphidicolin-S<br>Arac-hs	A677T (polymerase domain): catalytic impairment of the enzyme

**A314T** (37-42, 45)	VACV<br>CMLV<br>MPXV<br>CPXV	HPMPC<br>cHPMPC<br>HPMPA<br>HPMPDAP<br>HPMPO-DAPy	Higher resistance than A314V	HPMP-5-azaC-R<br>3-deaza-HPMPA-R	PMEO-DAPy-hs<br>PAA-hs<br>Aphidicolin-S<br>Arac-S	A314T (3′-5′ exonuclease domain, beta-hairpin): modulation of guidance from elongation to editing mode (45)
**A314V** (37-42, 45)	VACV<br>CMLV<br>MPXV<br>CPXV	HPMPC<br>HPMPDAP	Low resistance	HPMP-5-azaC-S<br>3-deaza-HPMPA-S	PMEO-DAPy-hs<br>PAA-hs<br>Aph-hs<br>AraC-hs	
**A314T + A684V** (37)	VACV	HPMPC<br>HPMPDAP	~ resistance as A314T	HPMP-5-azaC-R<br>3-deaza-HPMPA-S	PMEO-DAPy-S<br>PAA-S<br>Aphidicolin-S<br>Arac-S	Combination of mutations
**A314T + L696S**	VACV	HPMPDAP	~ resistance as A314T	HPMP-5-azaC-R<br>3-deaza-HPMPA-S	PMEO-DAPy-hs<br>PAA-hs<br>Aphidicolin-S<br>Arac-S	Combination of mutations<br>**L696S** (polymerase domain): catalytic impairment of the enzyme
**A314T + A684V + L696S**	VACV	HPMPDAP	Higher resistance than A314T + L696S	HPMP-5-azaC-R<br>3-deaza-HPMPA-R	PMEO-DAPy-R<br>PAA-S<br>Aphidicolin-S<br>Arac-S	Combination of mutations
**A314T + M656I + A684V**	VACV	HPMPC	Higher resistance than A314T + A684V	HPMP-5-azaC-R<br>3-deaza-HPMPA-S	PMEO-DAPy-hs<br>PAA-hs<br>Aphidicolin-S<br>AraC-hs	Combination of mutations<br>**M656I** (polymerase domain):
**A314T + T688A (37)**	VCAV	HPMPC	Resistance to ANPs	HPMP-5-azaC-R<br>3-deaza-HPMPA-S	PMEO-DAPy-hs<br>PAA-hs<br>AraC-hs<br>Aphidicolin-S	Combination of mutations<br>**T688A** (polymerase domain): indirect effect on template backbone binding (45)
**A314V + T500I**	VACV	HPMPDAP	~ A314V	HPMP-5-azaC-S<br>3-deaza-HPMPA-S	PMEO-DAPy-hs<br>PAA-hs<br>AraC-hs<br>Aph-hs	Combination of mutations<br>**T500I:** changes in enzyme structure
**A314V + T500I + T688A**	VACV	HPMPDAP	Higher resistance than A314V	HPMP-5-azaC-R<br>3-deaza-HPMPA-S	PMEO-DAPy-hs<br>PAA-hs<br>AraC-hs<br>Aph-hs	Combination of mutations

**A314V + A684V**	VACV	HPMPC	Higher resistance than A314V	HPMP-5-azaC-R<br>3-deaza-HPMPA-S	PMEO-DAPy-hs<br>PAA-hs<br>AraC-hs<br>Aphidicolin-hs	Combination of mutations
**A314V + L510S + A684V**	VACV	HPMPC	Higher resistance than A314V + A684V	HPMP-5-azaC-R<br>3-deaza-HPMPA-S	PMEO-DAPy-hs<br>PAA-hs<br>AraC-hs<br>Aph-hs	**L510S** (N-terminal domain): changes in enzyme structure
**Y332H**	VACV	PAA	Sensitive	HPMP-5-azaC-S<br>3-deaza-HPMPA-S	PMEO-DAPy-S<br>PAA-R<br>Aphidicolin-S<br>AraC-S	**Y332H** (3′-5′ exonuclease domain): Destabilization of the 3′-5‘exonuclease domain
**S338F (40)**	VACV	HPMPC	HPMPC-R - no data availale for other ANPs	Not available	Not available	**S338F** (3′-5′ exonuclease domain): indirect effect on complementary strand binding or on strand switching between elongation and editing mode (45)
**F354L**	VACV	PAA	Sensitive	HPMP-5-azaC-S<br>3-deaza-HPMPA-R	PMEO-DAPy-R<br>PAA-R<br>Aphidicolin-hs<br>AraC-R	**F354L** (DNA polymerase family B viral insert): altering the interaction between insert 2 and finger domain
**C356T**<br>G372D**<br>G380S** (44)**	VACV	PAA	Not available	Not available	PAA-R<br>Aphidicolin-hs<br>AraC-R +/-	**C356T, G372D, G380S**: (DNA polymerase family B viral insert): altering the interaction between insert 2 and finger domain (45)
**F171S + C356T<br>F171S + G372D<br>F171S + G380S (44)**	VACV	-	Not available	Not available	PAA-R<br>Aphidicolin-hs<br>AraC-R	Combination of mutations
**G372C**	VACV	PMEO-DAPy	Sensitive	HPMP-5-azaC-S<br>3-deaza-HPMPA-R	PMEO-DAPy-R<br>PAA-S<br>Aphidicolin-S<br>AraC-R	**G372C:** (DNA polymerase family B viral insert): altering the interaction between insert 2 and finger domain
**G392D (44, 53)**	VACV	*ts*	Not available	Not available	Not available	**G392D**: (DNA polymerase family B viral insert): desestabilization of insert 2 (45)
**R407C + L511F**	VACV	HPMPO-DAPy	Low resistance	HPMP-5-azaC-S<br>3-deaza-HPMPA-S	PMEO-DAPy-S<br>PAA-S<br>Aphidicolin-S<br>AraC-S	Combination of mutations<br>**R407C** (changes in enzyme structure)<br>**L511F** (changes in enzyme structure)

**M313I + R407C + L511F**	VACV	HPMPO-DAPy	Higher resistance than R407C + L511F	HPMP-5-azaC-S<br>3-deaza-HPMPA-S	PMEO-DAPy-S<br>PAA-hs<br>Aphidicolin-S<br>AraC-hs	Combination of mutations
**R155S + M313I + R407C + L511F + A684V**	VACV	HPMPO-DAPy	Higher resistance than M313I + R407C + L511F	HPMP-5-azaC-R<br>3-deaza-HPMPA-S	PMEO-DAPy-S<br>PAA-S<br>Aphidicolin-S<br>AraC-S	Combination of mutations
**D480Y**	VACV	HPMPDAP	Low resistance	HPMP-5-azaC-S<br>3-deaza-HPMPA-S	PMEO-DAPy-hs<br>PAA-S<br>Aphidicolin-S<br>AraC-hs	**D480Y** (3′-5′ exonuclease domain): indirect effect on complementary strand binding or on strand switching between elongation and editing mode
**D480D + A684V + A705T**	VACV	HPMPDAP	Higher resistance than D480Y	3-deaza-HPMPA-S<br>HPMP-5-azaC-R	PMEO-DAPy-S<br>PAA-R<br>Aphidicolin-hs<br>AraC-hs	Combination of mutations<br>**A705T** (palm domain): impaired catalytic activity
A498T<br>A498V* (44)	VACV	Aphidicolin	Not available	Not available	PAA-hs<br>Aphidicolin-R<br>AraC-hs & AraA-hs	**E498T/V:** Interference with the rotation of a base in the template requirred for aphidicolin binding (45)
E611K (44)	VACV	*ts*	Not available	Not available	Not available	**E611K:** Destabilization of insert 3 or of the elongation site (45)
L670M (44)	VACV	Aphidicolin	Not available	Not available	Aphidicolin-R	**L670M:** Indirect effect on the binding of the template backbone next to the aphidicolin-binding site (45)
**A684V (37, 39, 41, 42)**	VACV<br>CMLV<br>CPXV<br>MPXV	HPMPC<br>HPMPDAP<br>HPMPO-DAPy<br>PAA	Low resistance	HPMP-5-azaC-R<br>3-deaza-HPMPA-S	PMEO-DAPy-R<br>PAA-R<br>Aphidicolin-hs<br>AraC-S	**A684V:** Indirect effect on the binding of the template backbone in the elongation site (45)
**S686N**	VACV	*ts*	Not available	Not available	Not available	**S686N:** Destabilization of insert 3 or the elongation site (45)

**117Ins GSPD + E792D**	VACV	PAA	Sensitive	HPMP-5-azaC-S 3-deaza-HPMPA-S	PMEO-DAPy-R<br>PAA-R<br>Aphidicolin-hs<br>AraC-S	Combination of mutations<br>**117Ins GSPD:** (3′-5′ exonuclease domain): modifying the 3′ nucleotide binding at the exo-nuclease site<br>**E792D:** Modulation of domain movements by a modification at the thumb-palm domain con-nection and a changed interaction with the complementary strand
**T831I**	VACV	HPMPDAP	High resistance	HPMP-5-azaC-R<br>3-deaza-HPMPA-S	PMEO-DAPy-hs<br>PAA-hs<br>Aphidicolin: not available<br>AraC: not available	**T831I:** Modulation of domain movements by a modification at the thumb-palm domain con-nection and a changed interaction with the complementary strand (45)
**S851Y (41)**	VACV	HPMPDAP<br>HPMPO-DAPy<br>cHPMPA	Low resistance	HPMP-5-azaC-S<br>3-deaza-HPMPA-hs	PMEO-DAPy-hs<br>PAA-hs<br>Aphidicolin-S<br>AraC-hs	**S851Y:** Modulation of domain movements by a modification of the thumb-palm domain in-terface (45)
**S851Y + A353V**	VACV	cHPMPA	Higher resistance than S851Y	HPMP-5-azaC: not available<br>3-deaza-HPMPA-S	PMEO-DAPy-hs<br>PAA-hs<br>Aphidicolin-S<br>AraC-hs	**A353V:** (DNA polymerase family B viral in-sert): altering the interaction between insert 2 and finger domain
**S851Y + A632T**	VACV	HPMPO-DAPy	Higher resistance than S851Y	HPMP-5-azaC-R<br>3-deaza-HPMPA-S	PMEO-DAPy-hs<br>PAA-hs<br>Aphidicolin-S<br>AraC-S	Combination of mutations<br>**A632T** (polymerase domain): impaired cata-lytic activity
**S851Y + A684V (41)**	VACV	HPMPDAP	Higher resistance than S851Y	HPMP-5-azaC-R<br>3-deaza-HPMPA-R	PMEO-DAPy-S<br>PAA-R<br>Aphidicolin-S<br>AraC-S	Combination of mutations
**Ins851L**	CPXV	HPMPO-DAPy	Higher resistance than S851Y	HPMP-5-azaC-R<br>3-deaza-HPMPA-S	PMEO-DAPy-hs<br>PAA-hs<br>Aphidicolin-R<br>AraC-S	**S851Y** Modulation of domain movements by a modification of the thumb-palm domain inter-face (45)
**E856K**	VACV	PMEO-DAPy	Sensitive	HPMP-5-azaC-S<br>3-deaza-HPMPA-R	PMEO-DAPy-R<br>PAA-S<br>Aphidicolin-S<br>AraC-R	**E856K** (polymerase domain): impaired cata-lytic activity

**L924F**	CPXV	HPMPDAP	High resistance	HPMP-5-azaC-R<br>3-deaza-HPMPA-R	PMEO-DAPy-hs<br>PAA-hs<br>Aphidicolin-S<br>AraC-S	**L924F** (polymerase domain): impaired catalytic activity and/or effect on 3′-5′ exonuclease activity
**L924F + Ins408**	CPXV	HPMPDAP	~L924F	3-deaza-HPMPA-S<br>HPMP-5-azaC-R	PMEO-DAPy-hs<br>PAA-hs<br>Aphidicolin-S<br>AraC-S	**Ins408** (DNA polymerase family B viral insert): altering the interaction between insert 2 and finger domain

Abreviatons: ts (temperature sensitive), hs (hypersensitive), S (sensitive), R (resistance), NA: not available

* Mutator phenotype

** Antimutator
